# Hsp90 regulates the dynamics of its cochaperone Sti1 and the transfer of Hsp70 between modules

**DOI:** 10.1038/ncomms7655

**Published:** 2015-04-08

**Authors:** Alina Röhl, Daniela Wengler, Tobias Madl, Stephan Lagleder, Franziska Tippel, Monika Herrmann, Jelle Hendrix, Klaus Richter, Gordon Hack, Andreas B. Schmid, Horst Kessler, Don C. Lamb, Johannes Buchner

**Affiliations:** 1 Center for integrated protein science (CIPSM) at the Department Chemie, Technische Universität München, Lichtenbergstr. 4, 85747 Garching, Germany; 2 Department Chemie, Center for Nano Science, Center for integrated protein science (CIPSM) and Nanosystems Initiative München (NIM), Ludwig-Maximilians-Universität München, Butenandtstr. 5-13, 81377 München, Germany; 3 Institute of Molecular Biology & Biochemistry, Center of Molecular Medicine, Medical University of Graz, 8010 Graz, Austria; 4 Institute of Structural Biology, Helmholtz Zentrum München, Ingolstädter Landstrasse 1, 85764 Neuherberg, Germany; 5 Department Chemie, Institute for Advanced Study (IAS), Technische Universität München, Lichtenbergstr. 2a, 85747 Garching, Germany; 6 Department of Chemistry, Faculty of Science, King Abdulaziz University, 21589 Jeddah, Saudi Arabia

## Abstract

The cochaperone Sti1/Hop physically links Hsp70 and Hsp90. The protein exhibits one binding site for Hsp90 (TPR2A) and two binding sites for Hsp70 (TPR1 and TPR2B). How these sites are used remained enigmatic. Here we show that Sti1 is a dynamic, elongated protein that consists of a flexible N-terminal module, a long linker and a rigid C-terminal module. Binding of Hsp90 and Hsp70 regulates the Sti1 conformation with Hsp90 binding determining with which site Hsp70 interacts. Without Hsp90, Sti1 is more compact and TPR2B is the high-affinity interaction site for Hsp70. In the presence of Hsp90, Hsp70 shifts its preference. The linker connecting the two modules is crucial for the interaction with Hsp70 and for client activation *in vivo*. Our results suggest that the interaction of Hsp70 with Sti1 is tightly regulated by Hsp90 to assure transfer of Hsp70 between the modules, as a prerequisite for the efficient client handover.

## Abstract (web summary)

The chaperones Hsp70 and Hsp90 are physically linked via the cochaperone Sti1/Hop, that has two binding sites for Hsp70. Here, Röhl *et al.* show that binding of Hsp90 changes the conformation of Sti1/Hop and determines to which site Hsp70 binds, perhaps facilitating transfer of client proteins from Hsp70 to Hsp90.

## Graphical abstract

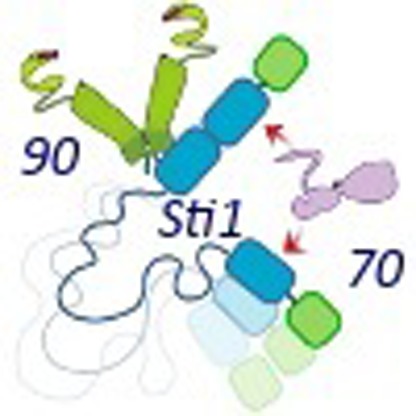

Hsp90 and Hsp70 are ubiquitous molecular chaperones. While Hsp70 associates with newly synthesized peptide chains, Hsp90 is required during later stages of folding and serves a more specialized set of client proteins^1^. Many of them are involved in signalling pathways, such as kinases and transcription factors^2^,^3^,^4^,^5^,^6^,^7^,^8^,^9^. For eukaryotic Hsp90, several cochaperones exist that stabilize specific conformations of Hsp90, which allows client activation^5^,^10^,^11^,^12^,^13^,^14^,^15^,^16^,^17^. The most common interaction site is the C-terminal EEVD motif of Hsp90 and Hsp70 that interacts with the TPR (tetratricopeptide repeat) domains of some cochaperones^18^. For efficient activation of some clients, Hsp90 and Hsp70 cooperate. The cochaperone Sti1 (stress-inducible protein 1, in mammals referred to as Hsp-organizing protein Hop)^19^,^20^ physically connects the two chaperones and regulates their activity thereby enabling the transfer of clients. Interestingly, Sti1/Hop is one of the few cochaperones substantially induced by stress^21^.

Sti1/Hop harbours three TPR domains, organized in two modules comprising one or two TPR domains followed by a DP (aspartate and proline rich) domain (Fig. 1a). The structures of the individual domains are known but their overall arrangement remains enigmatic^22^. The TPR domains are tandem repeats of a 34-amino-acid consensus motif, which forms a cleft of seven antiparallel alpha-helices capable of binding the C-terminal tails of Hsp90 and Hsp70 (ref. 23). In Sti1, the peptide-binding groove of TPR2A is highly specific for Hsp90. In addition, substantial contacts are formed between the Hsp90 M-domain and TPR2B^22^. Together, TPR2A–TPR2B stabilizes the open conformation of Hsp90 and inhibits its ATPase activity^22^,^24^,^25^,^26^,^27^. Simultaneous to Hsp90 binding, Sti1 is able to interact via TPR1 and TPR2B with Hsp70 (refs 22, 28, 29). However, it was unclear whether two Hsp70 molecules can contact TPR1 and TPR2B at the same time. Strikingly, deletion of TPR1 does not affect the ability of Sti1 to activate clients *in vivo*^22^. Therefore, the physiological relevance of the Hsp70-binding function of TPR1 remained unclear. Another intriguing feature of Sti1 is that each of the two Hsp70-binding TPR domains is followed by a DP domain. These DP domains, especially DP2 located C-terminally of the crucial TPR2B domain, are important for client activation *in vivo*^22^,^30^,^31^,^32^. Such a TPR–DP module is also present in Hip (Hsp70-interacting protein), an Hsp70 cochaperone^33^,^34^. Interestingly, although the two DP domains of Sti1 are structurally similar, DP2 cannot be exchanged by DP1 in terms of biological function^22^.

To resolve the molecular mechanism of Sti1, we analysed the structural dynamics of the modules in Sti1 and their interaction with Hsp70 and Hsp90. We show that Sti1 contains two functional modules separated by a linker. Binding of Hsp90 affects the interaction of Hsp70 with the two potential binding sites in Sti1.

## Results

### Sti1 is an elongated protein with a flexible hinge region

**Figure 1:**
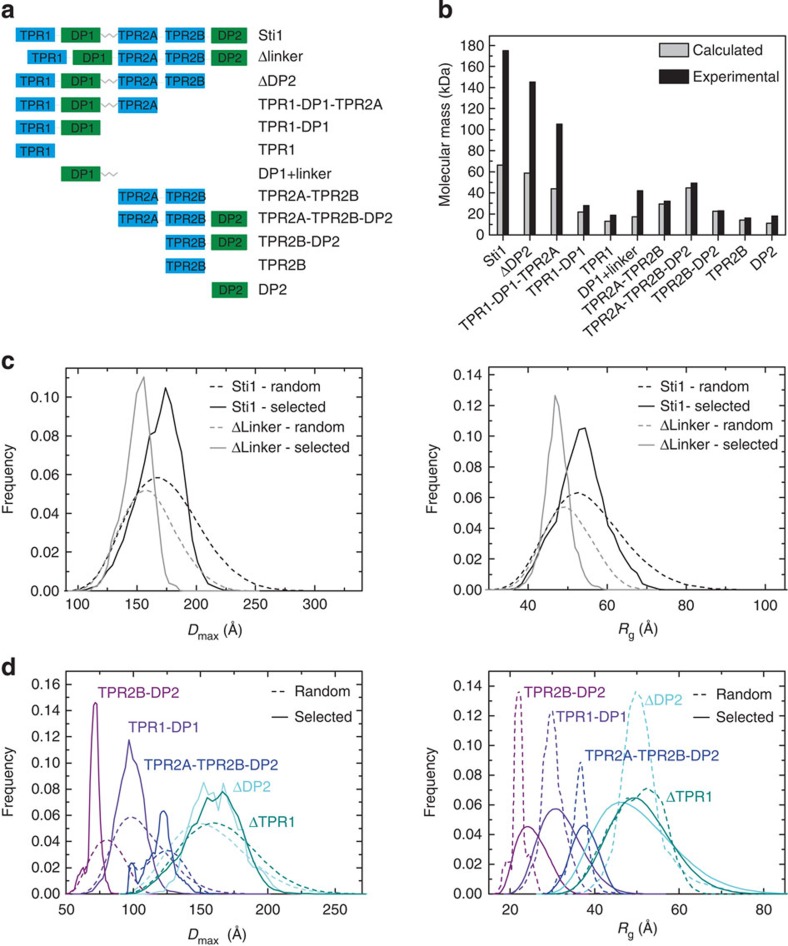
Overall shape of Sti1 and Sti1 fragments. (**a**) Scheme of Sti1 and Sti1 deletion variants investigated in this study. (**b**) Molecular mass of Sti1 variants as calculated assuming globular proteins (grey) and experimentally derived from analytical gel filtration experiments (black). The differences between these indicate that Sti1 fragments containing the linker exhibit an elevated hydrodynamic radius and possess an elongated shape in solution. (**c**,**d**) *D*_max_ (left) and *R*_g_ (right) distributions of the ensemble optimization method models of wild-type Sti1 and mutants in the initial pool of structures with randomized interdomain linkers (dashed lines) and in the selected ensembles (solid lines).

To obtain insight into the arrangement of the domains in full-length Sti1, we performed analytical gel filtration experiments and single-pair Förster Resonance Energy Transfer (spFRET) experiments with wild-type Sti1 and various mutants. The molecular mass for the wild-type protein determined using analytical gel filtration was largely increased compared with the calculated values for globular proteins (Fig. 1a,b). As previous studies showed that Sti1/Hop is a monomer^35^, this indicates an elongated shape of Sti1. To confirm this, we performed analytical ultracentrifugation experiments with Sti1 and a Sti1 variant, lacking the linker region. The sedimentation data indicate that both proteins are monomeric. We obtained frictional coefficients of 1.80±0.05 and 1.76±0.06, respectively, suggesting that both are elongated. Solution-based spFRET experiments on constructs where the donor and acceptor molecules were fluorescently labelled near the C- and N-terminus of Sti1 (S2C-G588C, S2C-S523C or G131C-S523C) exhibited no appreciable FRET signal (Supplementary Fig. 1). This indicates that the separation of fluorophores is larger than ∼100 Å. These results are in agreement with an elongated shape of Sti1 as previously suggested^35^,^36^. Furthermore, it may also be an indication of flexibility between modules. The unusual diffusion behaviour was also observed for the constructs that contained the linker region connecting DP1 and TPR2A (ΔDP2, TPR1–DP1–TPR2A and DP1+linker). In all other cases (TPR1–DP1, TPR2A–TPR2B, TPR1, TPR2A–TPR2B–DP2, TPR2B–DP2, TPR2B and DP2), the calculated and experimental molecular weights correlated (Fig. 1b). Together, this indicates that the linker is responsible for the elongated and possible dynamic nature of Sti1. To further address the conformation of Sti1, we performed small-angle X-ray scattering (SAXS) analysis. Scattering curves recorded at several solute concentrations confirmed that Sti1 constructs containing the linker region adopt an extended conformation as we observed a high radius of gyration and a large maximum diameter (for Sti1 wild-type: *R*_g_=58 Å and *D*_max_=260 Å, Supplementary Fig. 2a,d). We analysed the conformational space sampled by Sti1 and fragments thereof using the ensemble optimization method (EOM)^37^. Here, a pool of independent structures based on the amino-acid sequence and available structural information is first generated. A genetic algorithm is then used to select an ensemble of structures that best describes the experimental SAXS data (for more details see the Methods section). We identified the best ensembles concerning the agreement of experimental and back-calculated data (Supplementary Fig. 2c,e, see Methods) and compared the *R*_g_ and *D*_max_ distributions of the random pool of structures with the selected ensembles (Fig. 1c,d). For full-length Sti1, the selected ensemble clearly shows a higher degree of compaction compared with the random pool (Fig. 1c). This suggests that there must be inter-domain interactions within Sti1 that lead to a partial compaction of the molecule. On the basis of the *R*_g_ and *D*_max_ distributions of Sti1 ΔTPR1, TPR2A–TPR2B–DP2, ΔDP2, Δlinker, TPR1–DP1 and TPR2B–DP2 (Fig. 1c,d; see also Table 1 and Supplementary Fig. 2c for the structural ensembles), we find that the compaction is strongest within the TPR2B–DP2 module. The maxima of *R*_g_ and *D*_max_ distributions of constructs lacking the TPR2B–DP2 module (for example, ΔDP2, TPR1–DP1) is close to the maxima found for a random pool, and therefore compact conformations must be underrepresented in these ensembles. Deletion of the linker connecting DP1 and TPR2A (Δlinker) resulted in a substantial reduction of the molecular dimensions (*R*_g_=58.4 Å versus 50.1 Å; *D*_max_=264 Å versus 207 Å). In summary, these results show that the segment connecting TPR1 and DP1, as well as the linker between DP1 and TPR2A, is flexible and within these regions the Sti1 domains behave like beads on a string. In contrast, *R*_g_ and *D*_max_ distributions of constructs including the TPR2B–DP2 module (for example, ΔTPR1, TPR2A–TPR2B–DP2, Δlinker, TPR2B–DP2) show a shift towards lower *R*_g_ and *D*_max_ indicative of the presence of more compact conformations. In concordance with this, the *D*_max_ of TPR2A–TPR2B–DP2 and TPR2A–TPR2B is identical (both 130 Å; Supplementary Fig. 2a), indicating that the presence of the DP2 domain does not lead to an extension of the maximum diameter.

### TPR2A, TPR2B and DP2 of Sti1 form a rigid module

**Figure 2:**
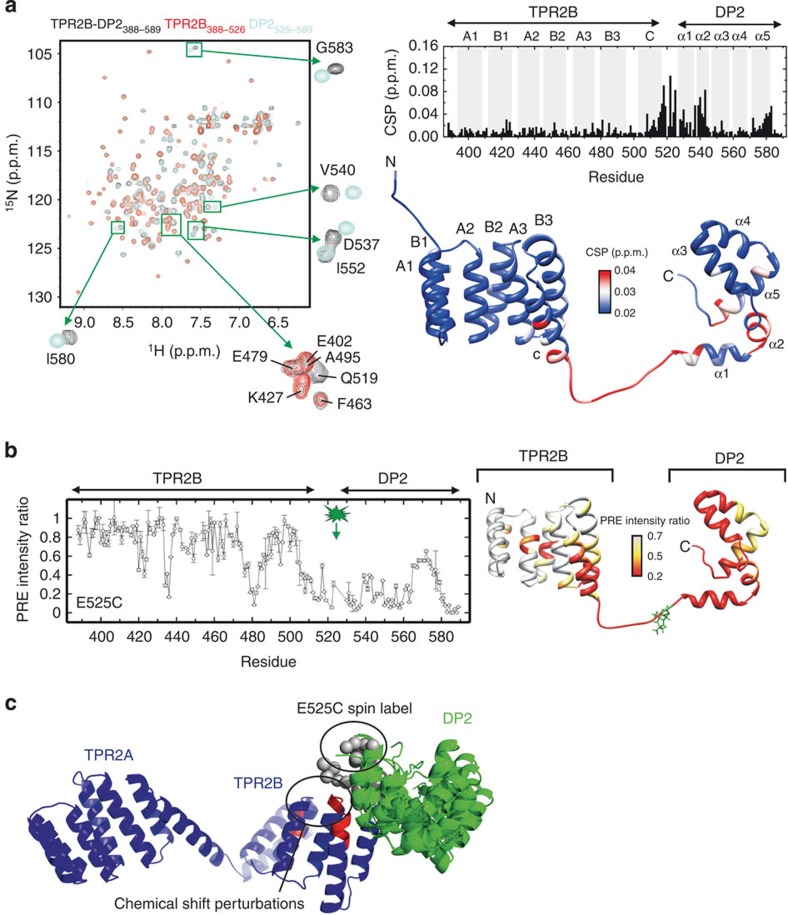
Interdomain contacts between TPR2B and DP2. (**a**) Left: overlay of ^15^N-HSQC spectra for isolated TPR2B, isolated DP2 and TPR2B-DP2. Right, upper panel: chemical shift differences plotted as a function against residues. Right, bottom panel: mapping of the shifts into a model of TPR2B-DP2 generated from the isolated structures (PDB 2LLW and 3UPV) using Xplor-NIH. (**b**) PRE data for the interaction between TPR2B and different PROXYL labelled DP2 variants in the two-domain construct and mapping of the positions onto a model of TPR2B-DP2. The position of the spin label is indicated in green. (**c**) NMR/SAXS model of Sti1 TPR2A–TPR2B–DP2. The five best structures based on the fit to the experimental data of 50 calculated structures were selected and aligned to TPR2A-TPR2B (residues 262–515). The positions of the E525C spin label, chemical shift perturbations and highest PREs in TPR2B (residues coloured red) are highlighted. The TPR and DP domains are shown in blue and green, respectively. The Cα atoms of flexible residues were modelled by the program CORAL and are shown as grey spheres.

As the SAXS data suggested that in the Sti1 TPR2A–TPR2B–DP2 segment DP2 is in contact with TPR2B, we compared NMR spectra of ^15^N-labelled TPR2B-DP2 with the spectra of the isolated domains (Fig. 2a). Shifts appeared not only for residues in the region connecting the two domains (residues 519 to 525) but also for residues in the DP2 domain (Fig. 2a). These are located in the first, second and the last helix of DP2 and also in the C-terminal end of TPR2B indicating an interaction between the two domains. We also performed paramagnetic relaxation enhancement (PRE) NMR measurements on TRP2B–DP2 constructs where cysteines were introduced at various positions within the DP2 domain (E525C, K536C, Q545C, N559C and N589C) and modified with a PROXYL spin label (Fig. 2b and Supplementary Fig. 3). Relaxation enhancement was mainly observed in DP2 but also residues in TPR2B and the linker connecting the two domains were affected. The strongest effects on TPR2B were observed when the spin label was attached to E525C (in the linker between TPR2B and DP2). Moreover, when the spin label was attached to a residue in the second helix of DP2 (K536C and Q545C), PRE effects were visible in the C-terminal helix of TPR2B and the linker region. Together with the chemical shift analysis data, these observations show that interdomain contacts are formed between the C-terminal helix of TPR2B, the linker and helix 1 and 2 of DP2.

To gain detailed insight into the organization of Sti1, we used spFRET as this method not only allows determination of distances on the molecular scale, but can also be used to investigate the flexibility of biomolecules. To look for dynamics within TPR2A–TPR2B–DP2 module, cysteines were introduced at positions G309C (TPR2A) and S523C (linker between TPR2B and DP2) (Supplementary Fig. 4a). The spFRET histogram has a single dominant peak with an FRET efficiency of∼20% and a donor–acceptor separation of∼65 Å (Supplementary Fig. 4b). A photon distribution analysis^38^ of the spFRET data indicates that the main FRET peak can be described by a single population with a Gaussian distribution exhibiting a s.d. of 5 Å. This indicates some flexibility between the FRET pair, but, considering that different domains were fluorescently labelled, this width is surprisingly narrow. In addition, no dynamic fluctuations were observed in the spFRET measurements on the TPR2A–TPR2B–DP2 module (Supplementary Fig. 4c). The idea of a rigidly connected TPR2B–DP2 segment derived from the spFRET experiments is in excellent agreement with the structural data, which also suggest that the orientation of DP2 with respect to TPR2B is fixed. In our structural model, DP2 is in contact with the last three α-helices of TPR2B and does not interfere with the Hsp70/Hsp90 peptide-binding site (Fig. 2c). In summary, our analysis shows that Sti1 is an elongated molecule in which a rigid C-terminal module consisting of three domains (TPR2A–TPR2B–DP2) cooperates with a two-domain N-terminal module (TPR1–DP1).

### Sti1 has two differentially activated Hsp70-binding sites

**Figure 3:**
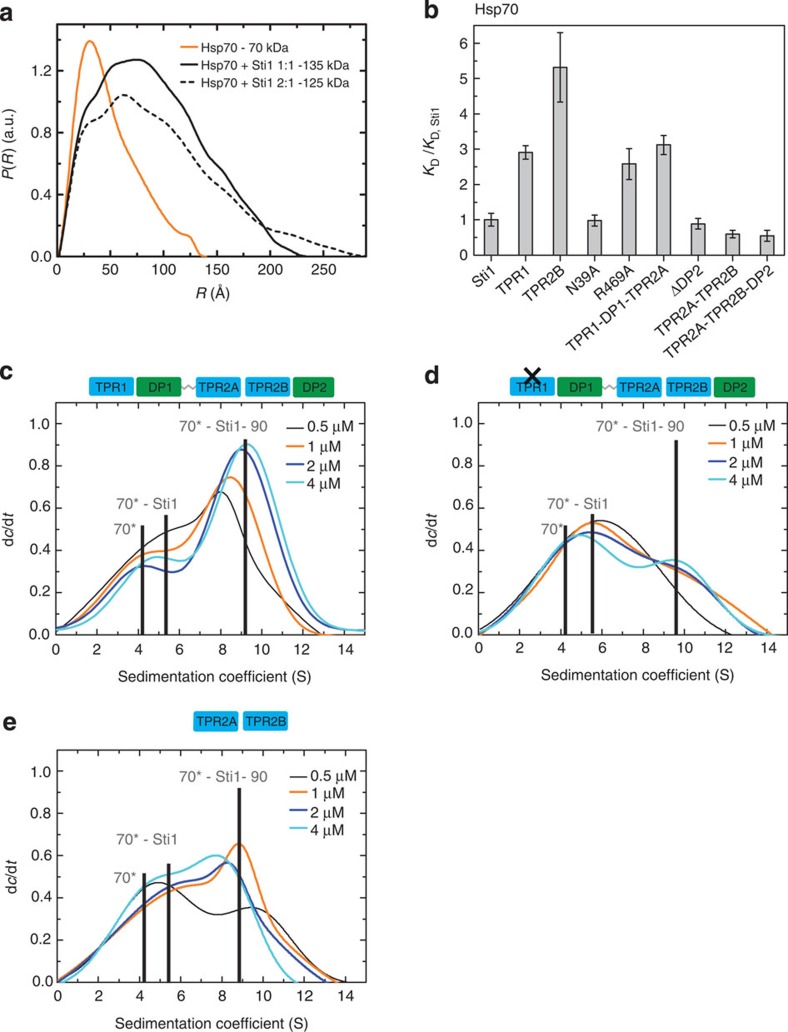
Hsp70-binding sites of Sti1. (**a**) SAXS data showing a comparison of the experimental radial density distributions of Hsp70 at increasing stoichiometric ratios of wild-type Sti1 as indicated. (**b**) Affinities of Sti1 variants towards yHsp70 determined by surface plasmon resonance spectroscopy with an Hsp70-coupled chip, normalized to wild-type Sti1 levels. Error bars indicate fitting error. (**c**–**e**) Formation of ternary Hsp90–Sti1–Hsp70 complexes using (**c**) wild-type Sti1, (**d**) Sti1 N39A and (**e**) Sti1 TPR2A-TPR2B. The 0.5 μM fluorescein-labelled yHsp70 and 3 μM yHsp90 were incubated with 0.5 (black), 1 (orange), 2 (blue) and 4 μM (cyan) Sti1 variant in 10 mM potassium phosphate at pH 7.5. Analytical ultracentrifugation was performed at 20 °C and 42 000 r.p.m. Sedimentation profiles were converted into d*c*/d*t* plots using standard procedures^46^ and fitted with bi-Gaussian functions. For clarity, only fits are shown, s.e. values were below 1%. As orientation, the lines indicate observed sedimentation coefficients (Hsp70* alone 4.2 S, left, binary complexes of Hsp70* with Sti1 5.6 S and ternary complexes of Hsp70* with Sti1 and Hsp90 8–10 S) from previous studies.

As demonstrated previously, Hsp70 can bind alternatively to TPR1 and TPR2B^22^. This is consistent with our SAXS analysis where Hsp70 is shown to bind TPR1 and TPR2B individually (Supplementary Fig. 2b,f). Our SAXS experiments also show that the molecular weight of the main complex between full-length Sti1 and Hsp70 does not change comparing a 1:1 to a 1:2 stoichiometry (Fig. 3a). As we used concentrations above both individual binding affinities, we conclude that full-length Sti1, although containing two Hsp70-binding sites, binds to Hsp70 in a 1:1 stoichiometry (Fig. 3a). This is in accordance with previous findings on the stoichiometry of the interaction of Hop and Hsp70 (refs 35, 36).

We further assessed the interaction of Hsp70 with Sti1 via surface plasmon resonance spectroscopy (SPR) analysis using an Hsp70-coupled chip (Fig. 3b). Both isolated TPR1 and TPR2B constructs specifically bound Hsp70 but exhibited weaker affinity towards Hsp70 compared with wild-type Sti1 (three- to fivefold), suggesting that additional interactions take place in the full-length construct. To better understand the role of the different domains for binding of Hsp70, we investigated full-length Sti1 constructs with specific mutations in the peptide-binding groove of the two TPR domains (mutation in TPR1: N39A, mutation in TPR2B: R469A) to disrupt the interaction with Hsp70 (Fig. 3b). These mutations were designed based on structural information on the interaction of the C-terminal peptide of Hsp70 and the respective TPR domain^22^. Surprisingly, the introduction of a TPR1-inactivating mutation (N39A) did not affect the affinity of Sti1 towards Hsp70. Likewise, fragments of Sti1 lacking TPR1 (TPR2A–TPR2B and TPR2A–TPR2B–DP2) exhibited similar dissociation constants. In contrast, mutation of the peptide binding groove of TPR2B (R469A) or complete loss of TPR2B (TPR1–DP1–TPR2A) reduced the affinity towards Hsp70 compared with wild-type Sti1. These results are consistent with both TPR1 and TPR2B being capable of binding Hsp70 in Sti1. However, the high-affinity interaction is mediated by the TPR2B domain and an intact TPR2A–TPR2B segment is necessary for the wild-type-like interaction with Hsp70.

To gain insight into the formation of ternary complexes, we employed analytical ultracentrifugation (AUC) (Fig. 3c–e). Sti1 variants were titrated to constant amounts of Hsp90 and fluorescently labelled yHsp70 (Hsp70*, Ssa1). In these experiments, generally three Hsp70-containing species can be observed: Hsp70* alone (4.2 S, left line), binary complexes of Hsp70* with Sti1 (5.6 S, middle line) and ternary complexes of Hsp70* with Sti1 and Hsp90 (8–10 S depending on saturation, right line). The first two complexes cannot be separated completely as the peaks in dc/d*t* plots overlap strongly. After addition of Sti1 to *Hsp70 and Hsp90, ternary complexes are formed at higher sedimentation coefficients (Fig. 3c), while in the absence of Sti1 *Hsp70 sediments as a single species with 4.2 S. At Sti1 concentrations between 0.5 and 4 μM more and more of the ternary complexes are formed, leaving only little amounts of free *Hsp70 observable at 4.2 S. With the TPR1-defective (Fig. 3d) as well as with the TPR1-lacking construct (Fig. 3e), ternary complexes were formed indicating that simultaneous binding of Hsp90 and Hsp70 occurs via binding to TPR2A–TPR2B. However, for these variants, ternary complex formation was weakened compared with wild-type Sti1. Taken together, these results emphasize that, while both TPR1 and TPR2B contribute to the interaction with Hsp70, TPR1 becomes more important as an Hsp70-binding site when Hsp90 is present. Furthermore, the data suggest that TPR1 and TPR2B are not independent in their interaction with Hsp70 but that there is communication between these domains in the context of Sti1.

### The Sti1 linker affects client activation *in vivo*

**Figure 4:**
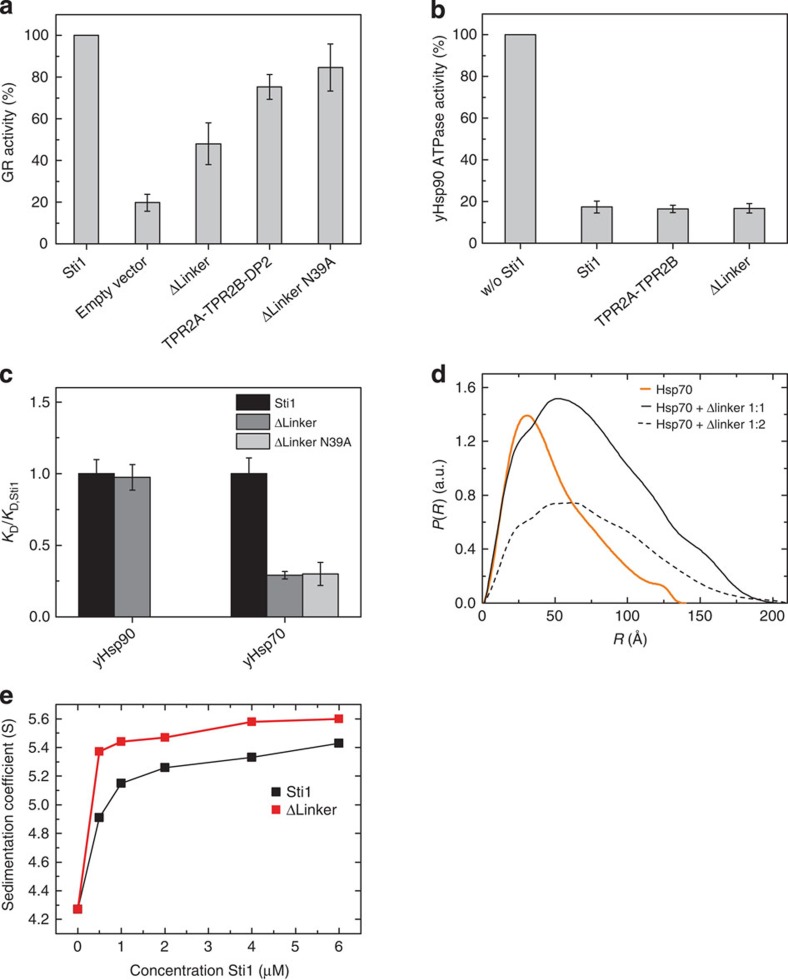
Effect of the linker deletion *in vivo* and on the interaction of Sti1 with Hsp70 and Hsp90. (**a**) Relative GR activity of ΔSti1 yeast cells expressing wild-type Sti1, Sti1 TPR2A-TPR2B-DP2, Sti1 Δlinker or Sti1 Δlinker N39A. GR activity of Sti1 wild-type expressing cells was set to 100%. Means of three independent experiments are shown. Error bars indicate s.e. values. (**b**) Inhibition of the yHsp90 ATPase activity by Sti1 and Sti1 linker deletion determined by an ATP-regenerative ATPase assay at 30 °C. Concentrations of 2 μM Hsp90, 2 μM Sti1 variant and 2 mM ATP were used. Means of three independent measurements are shown. Error bars indicate s.e. values (**c**) Binding affinities of wild-type Sti1, Sti1 Δlinker and Δlinker N39A with yHsp90 and yHsp70 determined by SPR. The binding affinities are normalized to Sti1 wild-type levels. Error bars indicate s.e. of the fit using titration data. (**d**) SAXS data showing a comparison of the experimental radial density distributions of Hsp70 at increasing stoichiometric ratios of Sti1 Δlinker. (**d**) Derived sedimentation coefficients from AUC runs using fluorescein-labelled yHsp70 with wild-type Sti1 or Sti1 linker deletion plotted against the concentration of the Sti1 variant (see Supplementary Fig. 5 for sedimentation profiles).

The two modules of Sti1 are connected to each other via a flexible linker. To investigate the biological function of this linker, we replaced it by a short Ser-Ala-Gly-Ala segment (Δlinker, Fig. 1a) and tested the effects of the linker deletion on the activation of the glucocorticoid receptor (GR), a stringent Hsp90 client, *in vivo*. A *S. cerevisiae sti1* knockout was transformed with plasmids for Sti1 variants, the GR and a β-galactosidase-based reporter system. In the absence of Sti1, GR activity was reduced to 20%. When the linker deletion mutant was expressed, the levels of activated GR decreased to about 50% of the levels observed for cells expressing wild-type Sti1 (Fig. 4a). Interestingly, client activation was even more reduced with the Δlinker mutant than was observed for the TPR2A–TPR2B–DP2 module alone where the TPR1–DP1 module and the linker have been completely removed (about 80% of wild-type Sti1) again, indicating communication between the two Hsp70 binding sites. Surprisingly, the negative effect of the linker deletion was abolished by the introduction of a point mutation in the peptide-binding groove of TPR1 (N39A), restoring GR activation to almost wild-type levels.

### The linker is important for Hsp70 interaction

To understand the role of the linker, we performed further *in vitro* analyses with the linker deletion. The stability and secondary structure of this mutant were comparable to wild-type Sti1 consistent with its unstructured character. In ATPase activity assays, the Sti1 Δlinker construct inhibited the ATPase of Hsp90 as efficient as wild-type Sti1 or the TPR2A–TPR2B fragment (Fig. 4b). Moreover, as shown by SPR analysis, the deletion of the linker did not affect the affinity of Sti1 towards Hsp90 (Fig. 4c). Hence, the linker deletion does not affect the Hsp90–Sti1 interaction.

Next, we assessed the interaction of the linker-deletion construct with Hsp70. SAXS experiments showed that the linker-deletion construct binds Hsp70 in a 1:1 stoichiometry (Fig. 4d) like wild-type Sti1. In AUC experiments, the Sti1 variants were titrated to constant amounts of labelled Hsp70. Complex formation with Sti1 could be observed by the peak shift to a maximum of about 5.6 S compared with 4 S for unbound Hsp70* (Supplementary Fig. 5). Surprisingly, the maximum sedimentation coefficient was already reached at lower concentrations of the linker-deleted variant compared with wild-type Sti1, indicating a tighter interaction with Hsp70 (Fig. 4e). SPR confirmed that the linker deletion increased the affinity for Hsp70 threefold (Fig. 4c). In the Sti1 Δlinker N39A construct where the binding of Hsp70 to TPR1 is impaired, no difference in binding affinity was observed compared with the Sti1 Δlinker mutant without the point mutation (Fig. 4c). Taken together, our findings suggest that conformations enabled by the linker interfere with the binding of Hsp70 to TPR2A–TPR2B in wild-type Sti1.

### The linker affects ternary complex formation

**Figure 5:**
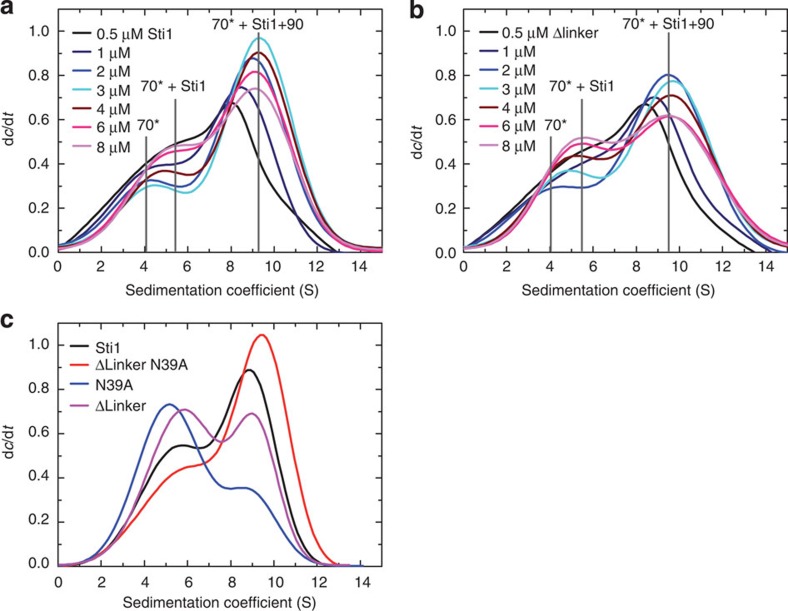
Deletion of the linker alters ternary complex formation. (**a**,**b**) Formation of ternary complexes between yHsp70, yHsp90 and either (**a**) wild-type Sti1 or (**b**) Sti1 Δlinker was visualized by analytical ultracentrifugation. The 0.5 μM fluorescein-labelled yHsp70 and 3 μM yHsp90 were incubated with 0.5 (black), 1 (navy), 2 (blue), 3 (cyan), 4 (wine), 6 (pink) and 8 μM Sti1 (light magenta) variant in 10 mM potassium phosphate at pH 7.5. Centrifugation was performed at 20 °C and 42,000 r.p.m. Sedimentation profiles were converted into d*c*/d*t* plots according to the standard procedures^43^ and fitted with bi-Gaussian functions. For clarity, only fits are shown. The s.e. values were below 1%. (**c**) Formation of ternary complexes between yHsp70, yHsp90 and wild-type Sti1 (black), Sti1 Δlinker N39A (red), Sti1 N39A (blue) and Sti1 Δlinker (pink) visualized by analytical ultracentrifugation. The 0.5 μM fluorescein-labelled yHsp70 and 3 μM yHsp90 were incubated with 3 μM Sti1 variant in 10 mM potassium phosphate at pH 7.5. Centrifugation was performed at 20 °C and 42,000 r.p.m. Sedimentation profiles were converted into d*c*/d*t* plots according to the standard procedures and fitted with bi-Gaussian functions. For clarity, only fits are shown. The s.e. values were below 1%.

To determine how the linker deletion affects the formation of ternary Hsp90–Sti1–Hsp70 complexes, we again performed AUC experiments using constant amounts of *Hsp70 and titrating in wild-type Sti1 or the linker-deleted Sti1 variant in the presence of unlabelled Hsp90 (Fig. 5a,b). Like before (see Fig. 3c), we observed more ternary Hsp90–Sti1–Hsp70 complexes (9.5 S) with increasing amounts of Sti1. At Sti1 concentrations higher than 3 μM, ternary complexes are reduced at the expense of *Hsp70-Sti1 complexes observable at 5.6 S. This suggests that there is now sufficient Sti1 to form individual binary complexes with *Hsp70 and Hsp90, thereby disrupting partly the ternary complexes. For the linker-deletion experiments, the formation of ternary complexes is reduced at the expense of *Hsp70-Sti1 binary complexes, which form at 5.6 S. While here also the amount of ternary complexes increased as long as Sti1 concentrations were below 3 μM, apparently not as much Hsp90 was incorporated into these complexes. Hence, *in vitro*, the population of binary and ternary complexes depends strongly on the ratio of the three components. Importantly, for the Sti1 Δlinker, the ternary complex was never as abundant and the binary complex was always more populated. This indicates that the deletion of the linker shifts the equilibrium of complexes towards higher amounts of binary complexes and a lower population of the ternary complex, which would indicate a weaker affinity for Hsp90.

In addition to the linker deletion, we introduced a mutation in TPR1 (N39A) and tested for ternary complex formation. Surprisingly, we detected increased ternary complex formation for this construct as compared with wild-type Sti1, even though the linker-deletion and N39A alone had the opposite effect (Fig. 5c). Together with the finding that a peptide-binding defective mutation in TPR2B enhances ternary complex formation^22^, this indicates that both Hsp70-binding sites contribute differently to ternary complex formation and that the effect of the linker on the formation of ternary complexes is strongly influenced by the interaction of Hsp70 with TPR1.

### Hsp70 and Hsp90 induce conformational changes in Sti1

**Figure 6:**
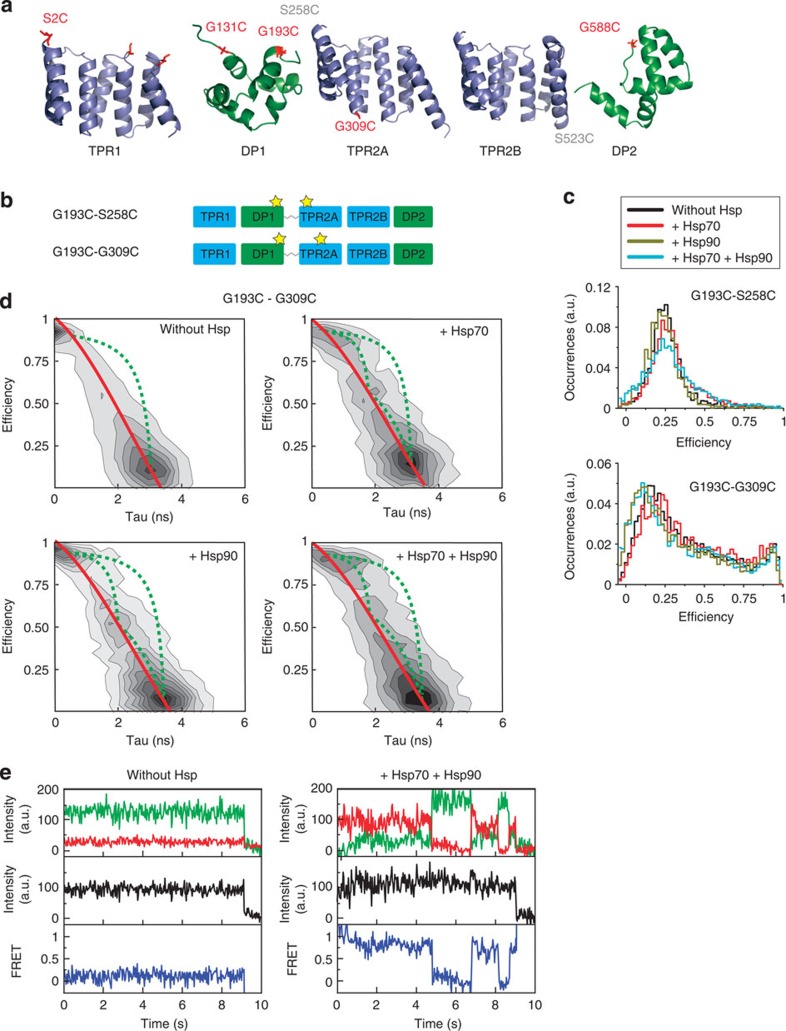
SpFRET measurements of fluorescently labelled Sti1. (**a**) The secondary structure of the different domains of Sti1. The residues mutated to a cysteine are shown in red (grey: not resolved in the structure). (**b**) Scheme of the different cysteine double mutants. The star marks the position of the fluorescent dye. (**c**) SpFRET efficiency plots of the mutants in panel (**b**). The 20 pM Sti1 was measured alone or mixed together with 10 μM Hsp90 or/and 25 μM Hsp70. The area under the curves was normalized. (**d**) FRET efficiency for the G193C-G309C mutant versus fluorescence lifetime of the donor in the presence of the acceptor. Multiple populations can be observed. The solid red line describes the expected relationship between donor lifetime and FRET efficiency when the populations are static, whereas the dashed green line indicates the theoretical curve when molecules undergo dynamic transitions between populations within a burst. In the absence of Hsps, the bursts fall symmetrically about the static line. In the presence of Hsps, a small deviation from the static line is observed for part of the population, suggesting there may be dynamic transitions between populations during a burst. (**e**) Representative data from spFRET TIRF experiments. Time traces of the donor and acceptor intensity (upper graph), total intensity corrected for the differences in sensitivity between the donor and acceptor channels (*I*_*T*_ =*γI*_*D*_+*I*_*A*_ where γ is the detection correction factor, middle graph) and FRET efficiency (lower graph) of the G193C–G309C Sti1 mutant in the absence and presence of Hsp70 and Hsp90. Left: a representative spFRET trace for the G193C–G309C Sti1 mutant in the absence of Hsps. Most of the traces of Sti1 in the absence of Hsps show a static FRET efficiency until either the donor or acceptor molecule photobleaches. In this case, the acceptor fluorophore photobleached first. Right: representative spFRET traces for the G193C–G309C Sti1 mutant in the presence of 25 μM Hsp70 and 10 μM Hsp90. A dynamic FRET signal is detectable in a significant fraction of the measured molecules. The fluctuations in the donor and acceptor fluorescence intensities indicate changes in the FRET efficiency due to the movement of Sti1 between different conformations. The donor fluorophore photobleached before the acceptor molecule and the total intensity dropped to background levels (see also Supplementary Fig. 6).

The above results suggest that crosstalk between the two modules is mediated by the linker and that the linker is important for the function of Sti1. To investigate the flexibility of the linker and conformational changes induced by Hsp binding, we performed spFRET experiments on constructs where the FRET pair spanned the linker region (G193C–S258C, S2C–G309C, G131C–G309C and G193C–G309C) (Fig. 6a). When labelling the ends of the linker region (G193C–S258C), no significant changes in the peak of the spFRET distribution were observed on the binding of Hsp70 and/or Hsp90 (Fig. 6b,c). Interestingly, there is an increase in the width of the spFRET distribution on binding of Hsp70, or Hsp70 and Hsp90, which indicates that chaperone binding increases the flexibility of Sti1. For the other mutants, a significant conformational change in Sti1 to lower FRET values was observed in the presence of Hsp90, indicating an increase in the distance between the two dyes (Fig. 6c and Supplementary Fig. 1). A detailed analysis of the G193C–G309C construct revealed the presence of at least three different conformations (Fig. 6c,d). In one conformation, the distance between the labels on the DP1 and TPR2A domains is extended and a low-FRET signal is detected (low-FRET conformation with a FRET efficiency of 15%). A second high-FRET conformation (*E*=95%) is also observable where the two domains are close to each other. A third intermediate-FRET conformation can be seen at *E*∼50%, particularly in the presence of Hsp70 or Hsp90 or both Hsps. Together this suggests that different conformations of Sti1 exist and that Hsp90 and Hsp70 modulate the equilibrium between these conformations.

### SpFRET experiments visualize dynamics of the Sti1 complex

A plot of the FRET efficiency determined using fluorescence intensity versus the fluorescence lifetime of the donor reveals the dynamics between the different conformations (Fig. 6d)^39^. For spFRET measurements with multiple static populations, the populations should follow the static line (shown in red in Fig. 6d). For Sti1 in the absence of Hsps, the distribution of FRET efficiency versus donor lifetime is symmetrically distributed around the static curve. The data are not scattered about the static line but deviates to the right for measurements performed with Hsp70 and/or Hsp90, suggesting the presence of dynamic fluctuations between the different conformations (dashed green lines in Fig. 6d). Fluctuations between different conformations on the millisecond to second timescale can be directly measured using spFRET on immobilized samples providing complementary information to the FRET histograms discussed above. Here, we encapsulated single Sti1 molecules with or without Hsps in vesicles, which were immobilized on the surface of quartz prisms (see Materials and Methods). To verify that encapsulation had no deleterious effects on the sample, burst analysis experiments of Sti1 in vesicles were compared with measurements of Sti1 without encapsulation and no significant differences were observed. Figure 6e shows exemplary surface-based spFRET traces for the G193C–G309C Sti1 construct alone and in the presence of Hsp70 and Hsp90 (also see Supplementary Fig. 6). In all cases, a fraction of molecules exhibited dynamics. For Sti1 alone, only a low percentage of traces showed dynamics (11%, Table 2). Upon the addition of Hsp70 or Hsp90, the percentage of dynamic molecules increased from 11% up to ∼25%. When both Hsps were present, we observed Sti1 switching between different conformations in up to ∼30% of the detected molecules. This suggests that binding of Hsp70 and/or Hsp90 to Sti1 induces dynamics in the protein that brings the TPR1 and TPR2 modules together.

To support this hypothesis, we performed surface-based spFRET experiments on Sti1 constructs with Hsp70-binding mutations in TPR1 (N39A) and TPR2B (N435A). For Sti1 alone or for Sti1 in the presence of Hsp90, the two Hsp70-binding mutants showed the same percentage of dynamics compared with Sti1 G193C–G309C (Table 2). Also for the G193C–G309C N39A construct, a similar increase in fraction of molecules exhibiting dynamics was observed in the presence of Hsp70 as with Hsp90 or with both chaperones. In contrast, the fraction of molecules showing dynamics for the G193C–G309C N435A mutant did not significantly change compared with Sti1 alone on addition of Hsp70 (15 versus 17%, respectively). Hence, binding of either chaperone to the TPR2A–TPR2B–DP2 module is sufficient to induce dynamics, but not binding of Hsp70 to TPR1.

Interestingly, when both Hsp70 and Hsp90 are bound, the two TPR mutants exhibited an increase in the fraction of dynamic molecules but this fraction was significantly less than that for wild-type Sti1. This again suggests that, for the ternary complex, both Hsp70-binding sites are important.

Kinetic information was extracted from the spFRET traces using a Hidden Markov model (HMM) analysis^40^ (Tables 3 and 4). Three FRET states were detected with FRET efficiencies of∼20% (low FRET), ∼60% (intermediate FRET) and ∼90% (high FRET). Transitions between all FRET states were observed indicating that all conformations can interconvert (Supplementary Fig. 7). This is consistent with the burst analysis experiments (Fig. 6d). For the G193C–G309C mutant, the kinetic rates were similar in the presence of Hsp70 and/or Hsp90. When the interaction of Hsp70 with either TPR1 or TPR2B is weakened by a mutation, the kinetic rates for several of the transitions increased when both Hsp70 and Hsp90 were present. The faster dynamics was also observed for Hsp70 alone in the G193C–G309C N435A mutant, whereas, as expected, the kinetics of all three constructs were similar in the presence of Hsp90 alone. Taken together, dynamics between at least three conformations of Sti1 were observed in the presence of Hsp70 and/or Hsp90, and all conformations can interconvert. In addition interactions of Hsp70 with both TPR1 and TPR2B decrease the rate of the dynamic transitions.

## Discussion

Sti1/Hop connects Hsp90 and Hsp70, the two major chaperone machineries in the eukaryotic cell^19^,^20^. Our analyses reveal that Sti1 consists of two structured modules with defined functions connected by a flexible linker. Of special interest is the C-terminal TPR2A–TPR2B–DP2 module. We have shown previously that the two TPR domains of this module are tightly linked and form a structural and functional unit^22^. Here, we show that the DP domain is positioned in a defined orientation to the TPR domains. Thus, all three domains are strongly coupled and exhibit limited inter-domain flexibility. This is particularly important, as the DP2 domain has a significant impact on client activation *in vivo*^22^,^30^,^31^,^32^. In contrast, the N-terminal module consisting of the TPR1 domain followed by DP1 is not a rigid unit. Rather, these domains are flexible concerning their relative orientation. It is tempting to speculate that the interaction of TPR1–DP1 with client-bound Hsp70 may be different compared with TPR2B–DP2. The third element in Sti1 is the long linker between the two TPR modules. As expected, it is flexible and unstructured. It provides dynamics in the relative orientation of the TPR modules, which has significant functional consequences such as effects on the formation of complexes with Hsp70 and Hsp90. The fact that the flexible linker in Sti1 plays a regulatory role is reminiscent of what is known about the linker in Hsp90 that connects the N-domain with the M-domain. In Hsp90, this linker also provides regulatory sites that are proposed to modulate Hsp90 in a client- and cochaperone-dependent manner^41^. The deletion of the linker in Sti1 reduces the activation of the client GR by 50%. These effects result from the higher affinity of the Sti1 Δlinker construct for Hsp70, which may be due to the closer distance between the alternative Hsp70-binding sites in Sti1 or a change in the dynamics of the protein.

Taken together, our results suggest that the linker acts as a molecular rheostat that, together with Hsp90, fine-tunes the affinity of Hsp70 for Sti1. The Hsp70–Hsp90–Sti1 chaperone machine is highly dynamic and needs to assemble and disassemble in a timely and ordered manner. Thus, slight changes in the binding affinities have a significant impact on the *in vivo* performance of this molecular machine.

Both TPR–DP modules contain an Hsp70-binding site, TPR1 and TPR2B. The presence of two TPR domains that alternatively bind Hsp70 was puzzling, especially, as some Sti1/Hop homologues lack the TPR1–DP1 module^42^,^43^. Although TPR1 is implicated in the interaction with Hsp70 (refs 29, 44, 45), it seems functionally obsolete as the activation of clients *in vivo* is not affected by its deletion^22^. TPR2B represents the high-affinity interaction site for Hsp70 in the absence of Hsp90. However, we see pronounced effects of the TPR1 domain *in vitro* in the presence of Hsp90. Also, in constructs in which the linker was deleted, TPR1 had a negative effect on client activity. When TPR1 was inactivated in this construct, client activation was increased. These findings point to a functional interplay between the different elements of Sti1. Despite its low affinity, our experiments define TPR1 as an important binding site for Hsp70 in Sti1. SpFRET experiments revealed that Hsp90 induces a more open conformation of Sti1 in which the two TPR–DP modules are further apart, increasing the accessibility of TPR1. Moreover, Hsp90 binds to TPR2A–TPR2B with a large interaction surface including an interaction of TPR2B with the Hsp90 M-domains^22^. This reduces the accessible space for binding of Hsp70 to TPR2B and would favour the binding of Hsp70 to TPR1. This explains why in previous work a change in affinity for Hsp70 was observed in the presence of Hsp90 (refs 12, 36, 43).

The presence of two Hsp70-binding sites that are differentially activated and regulated suggests that Hsp70 switches between these two sites during client activation. Strikingly, we observed increased dynamics between the two Sti1 TPR-DP modules upon addition of Hsp90 and Hsp70. The induced dynamics often brings the two Hsp70-binding domains (TPR1 and TPR2B) into close proximity. This would facilitate the transfer of Hsp70 between TPR1 and TPR2B and explain the existence of the flexible linker/hinge region. The function of the linker seems tightly connected to the interaction with Hsp70 as the effects of the deletion of the linker (for example, the decreased formation of ternary complexes *in vitro* and the client activation *in vivo*) are reversed by additional mutation of TPR1. This implies that, in the linker-deleted Sti1, Hsp70 binds to TPR1 and this represents a ‘dead end' as transfer of Hsp70 to the productive binding site TPR2B is disabled.

Together, this supports the notion that TPR1–DP1 is the Hsp70-client-recruiter. Another potential function of TPR1–DP1 is the removal of Hsp70 from the Hsp90-binding module once the client is transferred from Hsp70 to Hsp90. On the one hand, this would make room for other cochaperones to bind to Hsp90 and further progression of the Hsp90 cycle, and, on the other hand, leads to a regeneration of the productive TPR2A–TPR2B–DP2 platform. It would explain the observed higher affinity of the linker deletion for Hsp70 as the TPR1-induced dissociation of Hsp70 from TPR2B is no longer possible.

**Figure 7:**
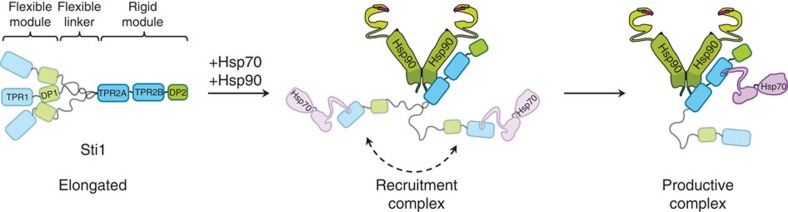
Schematic model of Sti1 dynamics regulated by Hsp90. Sti1 is an elongated protein comprising a flexible N-terminal module and a rigid C-terminal module connected by a central, flexible linker region. Following binding of Hsp70 and Hsp90, Sti1 exhibits dynamical behaviour, with the flexible linker mediating transitions between conformations in which the two modules are either apart or close in proximity. Such behaviour would facilitate an initial binding of Hsp70 to the Sti1-TPR1 domain in the open conformation, followed by rearrangement to the proximal conformation. From here, Hsp70 may be readily transferred to the Sti1–TPR2B domain, allowing it to come into close contact with Hsp90. This ‘productive' complex would then allow for transfer of the client protein between the two chaperones.

The presented results lead to a model for the mechanism by which Sti1 enables the transfer of clients from Hsp70 to Hsp90 (Fig. 7). Sti1 has an elongated structure with a flexible linker allowing interactions between the TPR1–DP1 and the rigid TPR2A–TPR2B–DP2 modules. Hsp90 binds to Sti1 mainly via the interaction with the C-terminal tail of Hsp90 to the peptide binding groove of TPR2A. Additional interactions occur between Hsp90-M and TPR2B. In this scenario, Hsp70 will preferentially bind to TPR1, which is flexibly connected to the rest of the protein. Conformational rearrangements mediated by the linker allow an approaching of the two Hsp70-binding sites, TPR1 and TPR2B. The close proximity of the Hsp70-binding sites facilitates the transfer of Hsp70 from TPR1 to TPR2B. The ternary complex allows client transfer and activation. After the client is transferred to Hsp90, TPR1–DP1 (potentially) removes Hsp70 from TPR2B for further progression of the chaperone cycle.

## Methods

### Protein purification

For Sti1 variants and yHsp90 (Hsp82), pET28 vectors carrying the respective genes plus an N-terminal 6 × His-SUMO-tag or a Thrombin cleavable 6xHis-tag were transformed into the *E. coli* strain BL21 (DE3) Codon Plus. Protein expression was induced at OD_600_ of 0.5 by addition of 1 mM isopropyl-β-D-thiogalactopyranosid (IPTG) overnight at 30 °C. In the case of full-length yHsp70, *Pichia pastoris* strain KM71H-Ssa1 (aox1::ARG4; arg4; *6xHis-SSA1* gene genomically inserted at AOX1 locus) was used for expression that was induced with 0.5% (v/v) methanol. Proteins were first purified by a 5-ml Hi-Trap column (GE Healthcare). After cleavage of the His_6_-SUMO tag or His-tag, respectively, gel filtration chromatography was performed with a Superdex 200 PrepGrade column (GE Healthcare) pre-equilibrated in 40 mM Hepes (pH 7.5), 150 mM KCl, 5 mM MgCl_2_. Point mutations of Sti1 were generated using the QuikChange II Site-Directed Mutagenesis Kit (Agilent).

### Fluorescent protein labelling

For spFRET experiments, native cysteines were removed and different double-cysteine mutants were generated such that the donor and acceptor fluorophores could be labelled at specific locations. The Sti1 double-cysteine variants were labelled stochastically at the cysteines with ATTO 532 and ATTO 647 (AttoTec) with a threefold excess of labels in 40 mM Hepes (pH 7.5), 150 mM KCl, 5 mM MgCl_2_ for 1 h at room temperature. The reaction was quenched with a 10-fold excess of DTT, and free label was separated from the protein on a Superdex 200 10/300 GL HPLC column (GE Healthcare). The cysteine mutations and fluorescent labelling did not alter the functionality of Sti1 (Supplementary Fig. 1d).

### ATPase assay

ATPase activities were measured using a regenerating ATPase assay as described before^25^. In this coupled enzyme assay, the reduction of NADH is detected by the decrease of absorbance at 340 nm using a Cary 50 Bio UV/VIS spectrophotometer (Varian). The assays were performed in 50 mM Hepes (pH 7.5), 50mM KCl, 5mM MgCl_2_ and 2 mM ATP. ATPase activity was measured at 30 °C using 2 μM yHsp90 and 2 μM Sti1 variant in a total volume of 150 μl. The ATPase reaction was started by adding 2 mM ATP. 100 μM Radicicol (Sigma Aldrich) was used to inhibit Hsp90 ATPase. The remaining ATPase activity in the presence of Radicicol was subtracted from the total activity.

### Analytical gel filtration

Experiments were performed with a Superdex 200 10/300 GL column (GE Healthcare) equilibrated in 40 mM Hepes (pH 7.5), 150 mM KCl, 5 mM MgCl_2_ using a flow rate of 0.8 ml min^−1^ at 25 °C. Signals were detected with a Jasco FP 920 fluorescence detector, using an excitation wavelength of 280 nm and an emission wavelength of 340 nm. Sti1 fragments were injected at concentrations from 0.2 to 20 μM.

### Analytical ultracentrifugation

yHsp70 (Ssa1) was covalently coupled to the amine-reactive dye 5-(and -6)-carboxyfluorescein (Invitrogen) as recommended by the manufacturer. AUC was carried out in a Beckman ProteomeLab XL-A (Beckman) equipped with a fluorescence detection system (Aviv Biomedical) using fluorescently labelled Ssa1 at concentrations of 500 nM and unlabelled proteins at concentrations of 3 μM if not indicated differently. Sedimentation analysis was carried out at 42,000 r.p.m. in a TI-50 Beckman rotor (Beckman) at 20 °C in 10 mM potassium phosphate (pH 7.5). To determine the size of the complexes, the raw data were converted to d*c/*d*t* profiles as described before^46^ and then analysed by bi-Gaussian or tri-Gaussian functions.

The determination of the frictional coefficient for Sti1 and Δlinker Sti1 was based on sedimentation experiments. The protein was detected using an absorbance optical system at 280 nm. Data analysis was performed using the c(S)-modul of UltraScan^47^.

### GR activity assay in yeast cells

The GR activity assay was performed as described previously^22^. In brief, the Δ*sti1* yeast strain YOR027w (BY4741; *Mat a*; *his3Δ1*; *leu2Δ0*; *met15Δ0*; *ura3Δ0*; YOR027w::kanMX4, from Euroscarf) was transformed with the human glucocorticoid receptor (hGR) expression vector (p413GPD-hGR), the reporter plasmid with GR-response elements pUCΔSS-26X and a p425GPD expression plasmid for Sti1 variants. Cells were treated with 10 μM desoxycorticosterone (DOX; Sigma Aldrich) for 8 h following determination of the β-galactosidase activity. Results are the mean of three independent experiments. Error bars indicate s.e.

### Surface plasmon resonance spectroscopy

SPR measurements were carried out with a BiacoreXTM instrument (GE Bioscience). Hsp70 or Hsp90 were covalently linked to a CM5 SPR chip deploying amine-coupling reagents, as described by the manufacturer. The analyte was flushed over the chip in 40 mM Hepes (pH 7.5), 20 mM KCl, 5 mM MgCl_2_, 0.005% (v/v) Tween with a constant flow of 10 μl min^−1^ at a temperature of 20 °C. The SPR signal change on injection was subtracted by the reference signal and plotted against the corresponding concentration. The direct binding signal was analysed by fitting the binding curves to a Langmuir absorption isotherm, deriving binary binding parameters.

### NMR

NMR spectra were recorded on a Bruker DMX600 or DMX750 spectrometer (BrukerBiospin) in 50 mM potassium phosphate (pH 7.5), 50 mM KCl, 1 mM TCEP. Data acquisition and processing was performed with Topspin 1.3 supplied by the manufacturer. Sparky (T. D. Goddard and D. G. Kneller, SPARKY 3, University of California, San Franscisco) was used for data evaluation. Calculation of the TPR2B-DP2 model based on the individual structures of the TPR2B and DP2 domains was done using XPLOR-NIH^48^ applying standard protocols. Spin labelling was performed by adding a twofold excess of (iodoacetamido)-proxyl (PROXYL, Sigma Aldrich) to single cysteine variants of Sti1. After removal of free label by a desalting column (GE Healthcare), the spin label was reduced by incubation with a 5- to 10-fold molar excess of ascorbic acid for 2 h at room temperature.

### Solution-based single-pair FRET experiments

For the single-pair FRET measurements, a home-built set-up with pulsed interleaved excitation and multiparameter fluorescence detection^49^ was used^50^. By using pulsed interleaved excitation-multiparameter fluorescence detection, we maximize the information gathered from the sample and can separate subpopulations based on fluorescence lifetime, anisotropy or labelling stoichiometry^51^. The fluorescently labelled Sti1 was diluted to a concentration <40 pM in 50 mM Hepes (pH 7.5), 150 mM KCl and 10 mM MgCl_2_ and measured alone or in the presence of 25 μM Hsp70 and/or 10 μM Hsp90. At 40 pM, the average molecule in the probe volume is much less than one. Analysis was performed with our in-house PAM software written in the MATLAB language (Mathworks). In the first step, all molecules with both dyes were selected and sorted in a FRET efficiency histogram. To look for the dynamic behaviour in the data, the lifetime of the donor was plotted versus the FRET efficiency. A molecule with no conformational changes can be described with the equation 
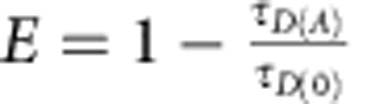
, where *τ*_*D*(*A*)_ is the lifetime of the donor in the present of the acceptor and *τ*_*D*(*0*)_ the lifetime of the donor in the absence of the acceptor. When the molecule switches between two states, the equation changes to 
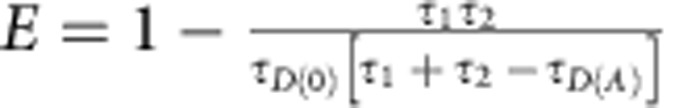
 where *τ*_1_ is the lifetime of the donor in one conformation and *τ*_2_ the lifetime of the donor in the second conformation^39^.

### Surface-based FRET experiments

For the analysis of dynamics of Sti1 on timescales larger than 30 ms, total internal reflection fluorescence microscopy (TIRF) was performed on proteins that were encapsulated in vesicles. We used vesicles to avoid artifacts of direct immobilization of the protein on the quartz prisms^52^. For preparing the vesicles, the lipids, which contain 300 μg 1,2-dioleoyl-sn-glycero-3-phosphocholine (DOPC, Avanti Polar Lipids) and 6 μg biotinylated lipid (1,2-dipalmitodyl-sn-glycero-3phosphoethanolamine-*N*-(cap biotinyl)(sodium salt)), were first mixed in chloroform. The chloroform was then removed by evaporation under a stream of nitrogen, and the remaining chloroform was removed in vacuum. The resulting films were hydrated for 30 min at room temperature with 200–340 nM fluorescently labelled Sti1 in buffer (50 mM Hepes (pH 7.5), 150 mM KCl and 10 mM MgCl_2_) and, when appropriate, also with 25 μM Hsp70 and/or 10 μM Hsp90. Multilamellar vesicles are formed in buffer and were extruded 31 times through polycarbonate membranes with a pore diameter of 200 nm using an extruder (Mini-Extruder Set, Avanti Polar Lipids) to make unilamellar vesicles with 200 nm diameter. To separate the vesicles from free protein, an Amicon spin column with a molecular weight cutoff of 100 kDa was used. In the last step, the purified vesicles were immobilized with a biotin-streptavidin-biotin linkage to the coating of PEG/3%-biotinylated PEG silanized to a quartz prism. The number of encapsulated proteins per volume follows a Poisson distribution. The concentration of Sti1 chosen was smaller than 400 nM so that the number of proteins per vesicle was less than one on average.

The spFRET TIRF measurements were performed on a home-built microscope as described previously^53^. A sample chamber was formed between the quartz prism and the coverslip with parafilm, where a channel was been cut. The sample was directly bound to the surface of the quartz prism. Excitation, using the 532-nm line of a diode-pumped solid-state laser (Cobolt Samba 532 nm, Cobolt), was performed through the prism and was illuminating the sample beyond the critical angle. The fluorescence of donor and FRET-sensitized acceptor emission from a single molecule was collected opposite the prism through the coverslip by a water immersion objective (CFI Plan Apochromat 60x WI, NA 1.2, Nikon) and was separated according to the spectral properties for the donor and acceptor dyes with a dichroic mirror. The donor and acceptor channels were imaged on different regions of an EMCCD camera (iXon +, Andor Technology) and spectrally mapped onto each other using a sample of fluorescent beads. After the mapping, the donor and acceptor signals per labelled molecule were further analysed with in-house software written in the MATLAB language. All intensity traces where more than one bleaching step was observed were discarded from the analysis.

For TIRF traces that exhibited dynamics, we analysed the kinetics using a Hidden Markov Model (HMM) analysis^40^. For each condition, the traces that showed clear dynamics were manually selected and fit to a global three-state HMM analysis. The software returned the FRET efficiencies of the three states found along with the corresponding width for each state. The results are plotted in Tables 3 and 4. From the values given by the HMM analysis, the Viterbi path was determined for each trace, which yields the most likely time course for the individual FRET traces. This makes it possible to determine the transition density plots for the different conditions, shown in Supplementary Fig. 7.

### SAXS

All SAXS data were recorded on an in-house SAXS instrument (SAXSess mc2,Anton Paar) equipped with a Kratky camera, a sealed X-ray tube source and a two-dimensional Princeton Instruments PI·SCX:4300 CCD detector (Roper Scientific). The scattering patterns were measured with 90 or 180 min exposure times (540/1080 frames, each 10 s) for several solute concentrations in the range from 1 to 10 mg ml^−1^. Radiation damage was excluded based on a comparison of individual frames of the 90/180-min exposures, where no changes were detected. A range of momentum transfer of 0.012<s<0.63 Å^−1^ was covered (*s*=4π sin(*θ*)/*λ*, where 2*θ* is the scattering angle and *λ*=1.5 Å is the X-ray wavelength).

All SAXS data were analysed with the package ATSAS (version 2.5). The data were processed with the SAXSQuant software (version 3.9), and desmeared using the programs GNOM^54^ and GIFT^55^. The forward scattering, *I*(0), the radius of gyration, *R*_g_, the maximum dimension, *D*_max_, and the inter-atomic distance distribution functions, (P(R)), were computed with the program GNOM. The masses of the solutes were evaluated by comparison of the forward scattering intensity with that of a human serum albumin reference solution (molecular mass 69 kDa). EOM calculations were carried out using the EOM program^37^ and using default settings. A random pool of 100,000 independent structures was generated using the primary sequence and the available structures of the individual domains as input (TPR1, DP1, TPR2A-TPR2B, DP2). The disordered regions in the termini and connecting domains were randomized. Using the built-in genetic algorithm and using the default settings, a subset of a few independent structures was selected that describes the experimental SAXS best and used to prepare the figures showing *R*_g_/*D*_max_ distributions and structural ensembles.

The structure of Sti1 TPR2A-TPR2B-DP2 was modelled using the program CORAL^56^. Inputs were the structures of TPR2A-TPR2B (PDB 3UQ3; note that, in solution, TPR2A-TPR2B adopts the same conformation as observed in the crystal structure as judged by the excellent agreement of experimental and back-calculated SAXS data—see Supplementary Fig. 2g) and DP2 (PDB 2LLW), SAXS data, NMR data for the TPR2B-DP2 binding interface (residues 508, 510–513 of TPR2B and residues 537–543, 572, 579–583 of DP2 are shifted (Fig. 2a) and must therefore be in the interface), and inter-domain PRE data (maximum distance of 20 Å between C^α^ atom of DP2 residue 525 and TPR2B residues 440 and 480; Fig. 2b). Only the most affected residues were used in the calculations due to the limited number of includable distance restraints in the program CORAL. Throughout the calculations, residues 516–524 connecting TPR2B and DP2 were defined as flexible and randomized. A total of 50 structures were calculated, and the best structures based on the fit to the experimental data selected to prepare Fig. 2c. For an overview of SAXS methods and data analysis, see Supplementary Table 1

## Author contributions

A.R., D.W., S.L., F.T., M.H., J.H., G.H., K.R. and A.B.S. performed experiments. A.R., H.K., J.B. and D.C.L. designed experiments. T.M. performed and analysed the SAXS experiments,. A.R., J.B. and D.C.L. wrote the manuscript. J.B. conceived the project.

## Additional information

**How to cite this article:** Röhl, A. *et al.* Hsp90 regulates the dynamics of its cochaperone Sti1 and the transfer of Hsp70 between modules. *Nat. Commun.* 6:6655 doi: 10.1038/ncomms7655 (2015).

## Supplementary Material

Supplementary Information Supplementary Figures 1-7 and Supplementary Table 1

## Figures and Tables

**Table 1:** SAXS data and analysis.

**Sample**	**Stoichiometry**	***R***_**g**_ **(Å)**	***D***_**max**_ **(Å)**	Molecular mass (kDa)*
Sti1	—	58.4±0.6	264	70
Sti1 Δlinker	—	50.1±0.5	207	60
Sti1–ΔTPR1	—	54.0±0.1	200	48
Sti1–TPR2A–TPR2B–DP2	—	36.0±0.1	130	35
Sti1–ΔDP2	—	60.3±0.1	220	58
Sti1–TPR1–DP1	—	30.4±0.1	100	29
Sti1–TPR2B–DP2	—	23.2±0.1	80	17
Sti1–TPR2A–TPR2B	—	33.9±0.1	130	24
Hsp70	—	40.0±0.1	140	70
Sti1+Hsp70	1:1	71.2±0.1	240	135
	1:2	76.2±0.1	280	123
Sti1 Δlinker+Hsp70	1:1	60.1±0.1	200	138
	1:2	59.7±0.1	200	100
Sti1–TPR1+Hsp70	1:1	45.0±0.2	160	86
Sti1–TPR2B+Hsp70	1:1	45.7±0.1	170	80

^*^The molecular mass was determined from the scattering intensity at zero angle (*I*(0)) using BSA as a reference.

**Table 2:** Percentage of dynamic traces in total internal reflection fluorescence microscopy measurements.

	**G193C–G309C dynamic traces (%)**	**G193C–G309C N435A dynamic traces (%)**	**G193C–G309C N39A dynamic traces (%)**
Without Hsp	17	17	13
+ Hsp70	28	15	27
+ Hsp90	31	26	27
+ Hsp70+Hsp90	28	21	23

**Table 3:** FRET efficiency values extracted from the HMM analysis of the TIRF measurements.

	**Low**	**Intermediate**	**High**
*G193C—G309C*			
+ Hsp70	0.16	0.52	0.84
+ Hsp90	0.24	0.66	0.92
+ Hsp70+Hsp90	0.23	0.57	0.86
			
*G193C—G309C N39A*			
+ Hsp70	0.18	0.62	0.92
+ Hsp90	0.10	0.58	0.91
+ Hsp70+Hsp90	0.14	0.58	0.91
			
*G193C—G309C N435A*			
+ Hsp70	0.18	0.65	0.93
+ Hsp90	0.20	0.57	0.92
+ Hsp70+Hsp90	0.17	0.57	0.92

FRET=Förster resonance energy transfer; HMM=Hidden Markov model; TIRF=total internal reflection fluorescence microscopy.

**Table 4:** Transition rates (in s^−1^) extracted from the HMM analysis of the TIRF measurements.

	**Low → high** **(s**^−1^**)**	**High → low** **(s**^−1^**)**	**Low → intermediate** **(s**^−1^**)**	**Intermediate → low** **(s**^−1^**)**	**Intermediate → high** **(s**^−1^**)**	**High → intermediate** **(s**^−1^**)**
*G193C–G309C*						
+ Hsp70	4.5 (83)	1.0 (49)	1.5 (64)	1.5 (69)	0.9 (48)	1.5 (53)
+ Hsp90	3.4 (36)	0.6 (35)	1.7 (79)	0.9 (69)	0.9 (22)	0.6 (22)
+ Hsp70+Hsp90	4.3 (51)	0.9 (41)	1.0 (22)	0.7 (29)	1.0 (34)	0.8 (35)
						
*G193C–G309C N39A*						
+ Hsp70	3.5 (31)	1.0 (19)	1.5 (20)	0.8 (19)	1.7 (23)	1.0 (18)
+ Hsp90	3.0 (52)	1.3 (27)	2.1 (28)	2.4 (36)	1.3 (43)	1.3 (39)
+ Hsp70+Hsp90	5.0 (15)	4.1 (7)	3.1 (22)	2.3 (27)	3.0 (8)	1.9 (7)
						
*G193C–G309C N435A*						
+ Hsp70	2.5 (20)	2.3 (8)	3.8 (18)	1.5 (14)	3.3 (6)	2.5 (3)
+ Hsp90	2.7 (10)	0.8 (9)	2.2 (23)	1.8 (25)	1.7 (26)	1.6 (13)
+ Hsp70+Hsp90	4.1 (32)	4.0 (19)	3.2 (26)	2.4 (26)	2.4 (19)	1.2 (19)

Abbreviations: HMM=Hidden Markov model; TIRF=total internal reflection fluorescence microscopy. The number of measured transitions is given in the parenthesis next to the respective rates.
