# Supplementary material for: Hsp90 regulates the dynamics of its cochaperone Sti1 and the transfer of Hsp70 between modules
Source: Nat Commun. 2015 Apr 8;6:6655. doi: 10.1038/ncomms7655 (PMC4403447; doi:10.1038/ncomms7655)
Supplement: Supplementary Information — Supplementary Figures 1-7 and Supplementary Table 1 [file ncomms7655-s1.pdf]

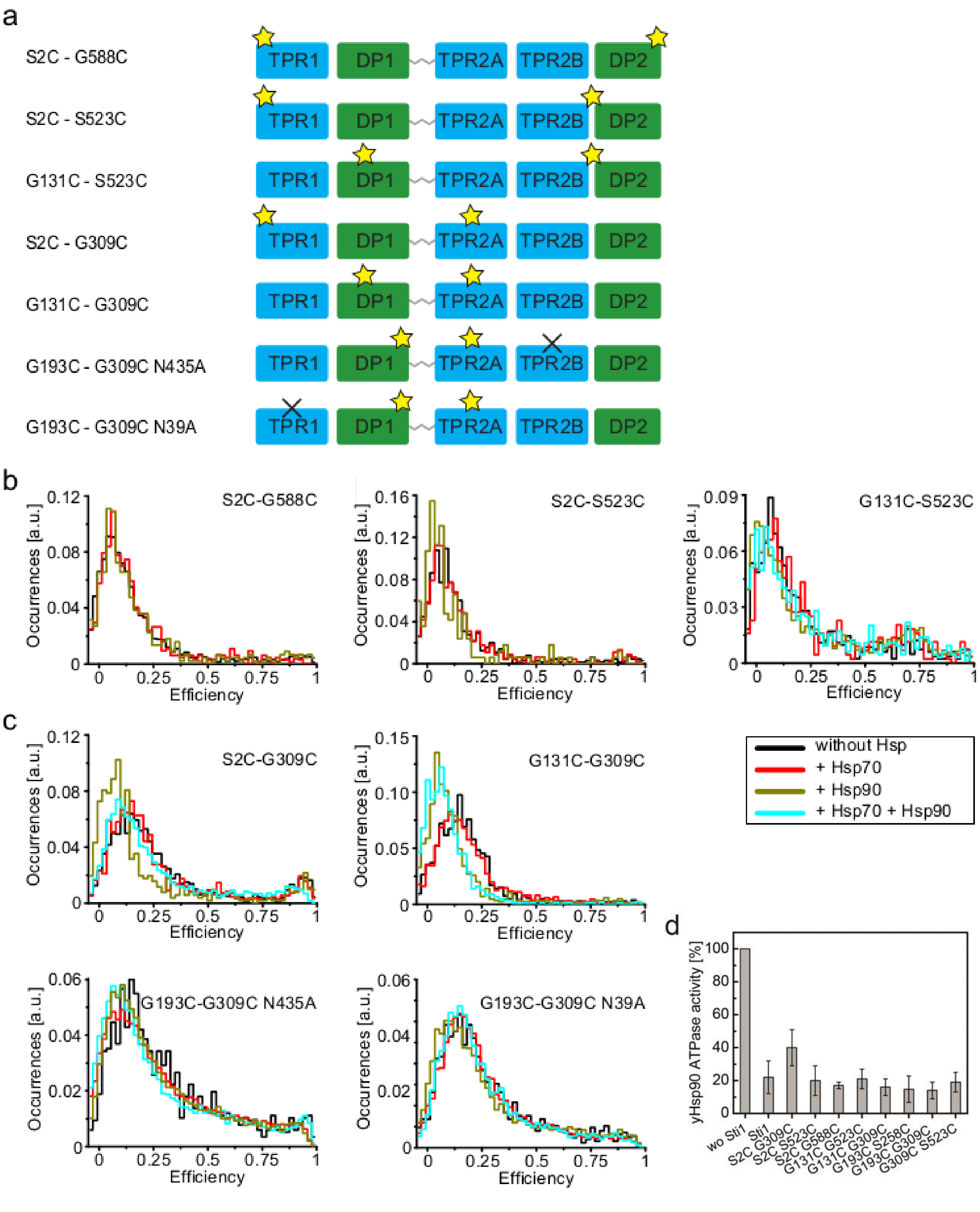
a
S2C - G588C
S2C - S523C
G131C - S523C
S2C - G309C
G131C - G309C
G193C - G309C N435A
G193C - G309C N39A
TPR1
DP1
TPR2A
TPR2B
DP2
b
S2C-G588C
S2C-S523C
G131C-S523C
Occurrences [a.u.]
Efficiency
c
S2C-G309C
G131C-G309C
G193C-G309C N435A
G193C-G309C N39A
without Hsp
+ Hsp70
+ Hsp90
+ Hsp70 + Hsp90
d
yHsp90 ATPase activity [%]
wo Sti1
Sti1
S2C G309C
S2C S523C
S2C G588C
G131C G523C
G131C G309C
G193C S258C
G193C G309C
G309C S523C

Supplementary Figure 1: SpFRET measurements of labeled Sti1. (a) Overview of the various double cysteine mutants discussed in this figure. The star marks the position of the cysteines, which were stochastically labeled with maleimide derivatives of ATTO532 and ATTO647. **(b-c)** SpFRET efficiency plots of the mutants in panel (a). **(b)** SpFRET histograms for labels near the ends of Sti1 investigating the overall conformation of the full-length protein. **(c)** SpFRET histograms for investigating the conformation of the linker. For all histograms, ~20 pM Sti1 was measured alone or mixed together with 10 µM Hsp90 or 25 µM Hsp70 or both. The area under the curves was normalized to one. **(d)** Effects of cysteine replacements on the functionality of Sti1. Hsp90 ATPase activity measured with an ATP-regenerating assay using 2 µM Hsp90 and 2 µM Sti1 and 2 mM ATP. Means of three independent measurements with the standard error are shown. Similar experiments were performed with labeled proteins which also resulted in wild-type similar ATPase activities.

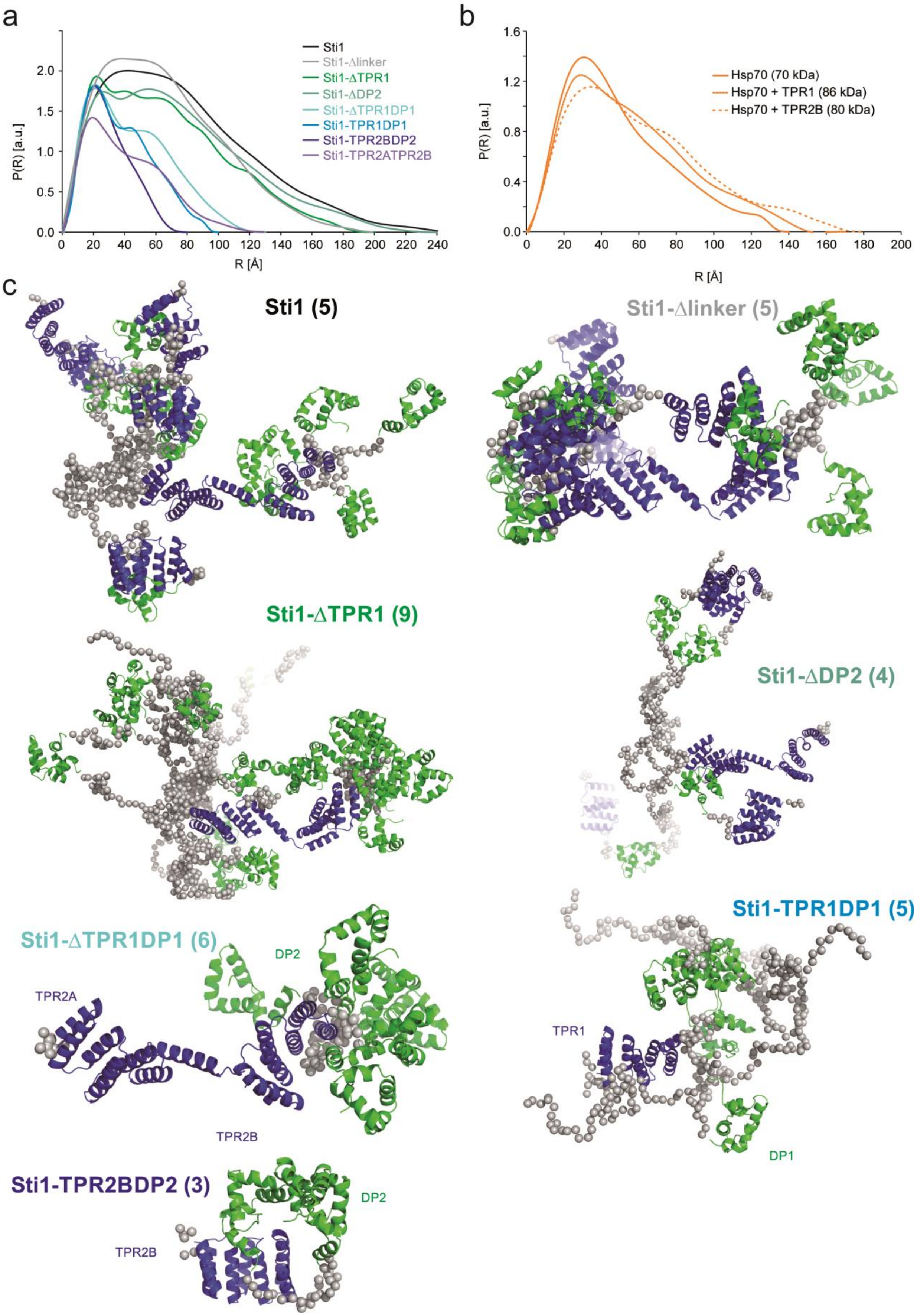
a
P(R) [a.u.]
R [Å]
Sti1
Sti1-Δlinker
Sti1-ΔTPR1
Sti1-ΔDP2
Sti1-ΔTPR1DP1
Sti1-TPR1DP1
Sti1-TPR2BDP2
Sti1-TPR2ATPR2B
b
Hsp70 (70 kDa)
Hsp70 + TPR1 (86 kDa)
Hsp70 + TPR2B (80 kDa)
c
Sti1 (5)
Sti1-Δlinker (5)
Sti1-ΔTPR1 (9)
Sti1-ΔDP2 (4)
Sti1-ΔTPR1DP1 (6)
DP2
TPR2A
TPR2B
Sti1-TPR1DP1 (5)
TPR1
DP1
Sti1-TPR2BDP2 (3)
DP2
TPR2B

d

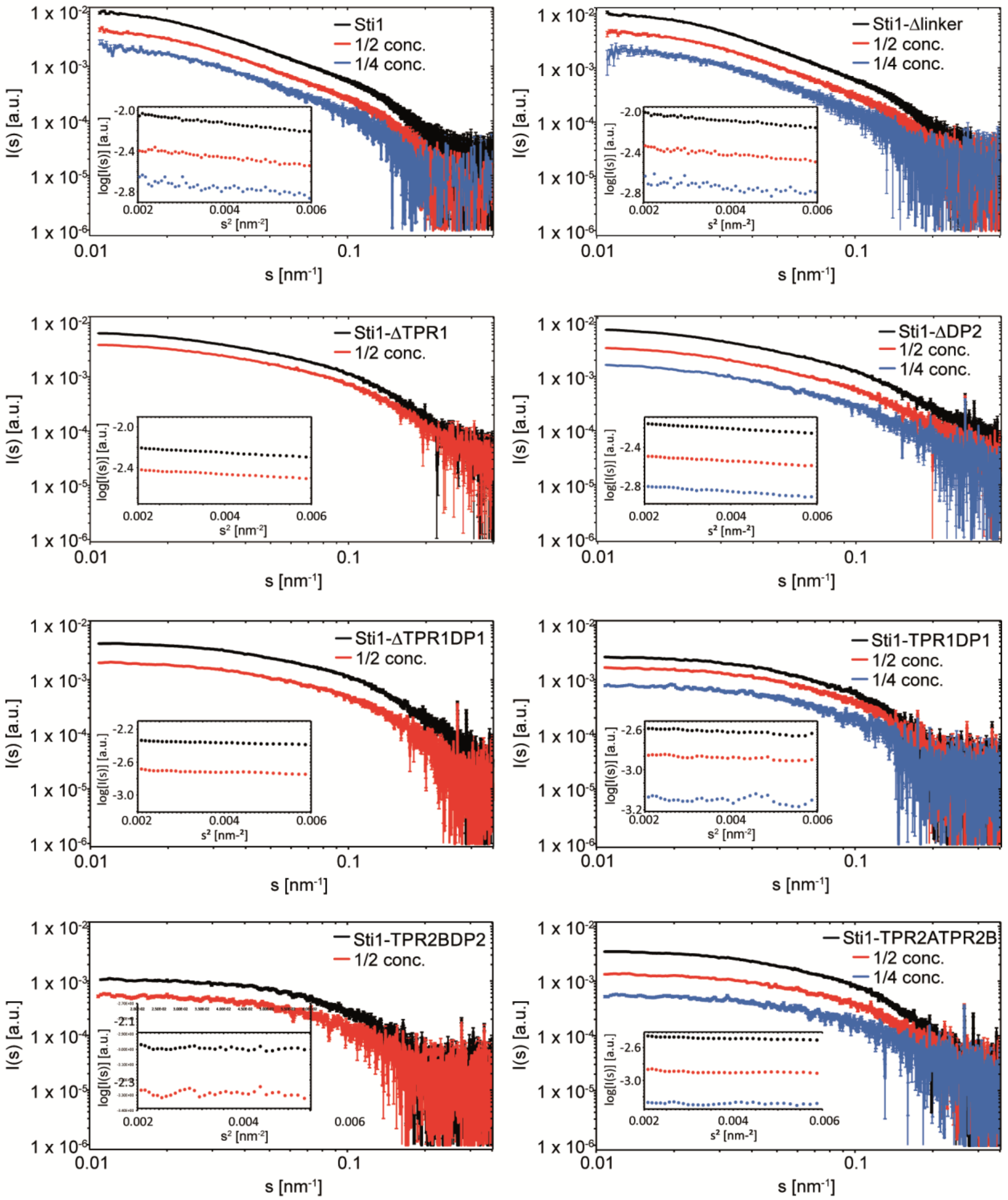
Sti1
1/2 conc.
1/4 conc.
Sti1-Δlinker
1/2 conc.
1/4 conc.
Sti1-ΔTPR1
1/2 conc.
Sti1-ΔDP2
1/2 conc.
1/4 conc.
Sti1-ΔTPR1DP1
1/2 conc.
Sti1-TPR1DP1
1/2 conc.
1/4 conc.
Sti1-TPR2BDP2
1/2 conc.
Sti1-TPR2ATPR2B
1/2 conc.
1/4 conc.
I(s) [a.u.]
s [nm-1]
log[I(s)] [a.u.]
s² [nm-²]
1 x 10-2
1 x 10-3
1 x 10-4
1 x 10-5
1 x 10-6
0.01
0.1
0.002
0.004
0.006

e

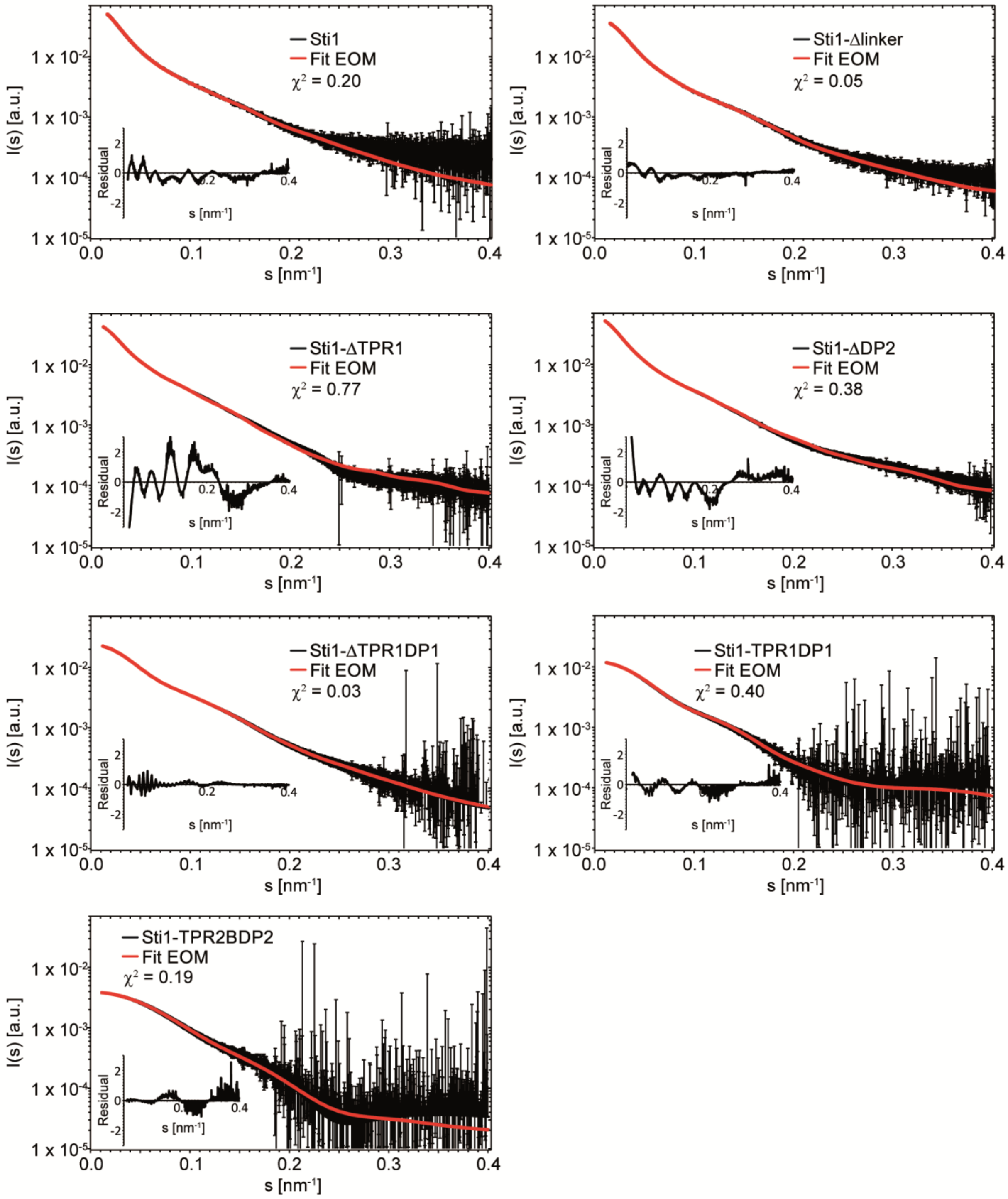
Sti1
Fit EOM
χ^2 = 0.20
Sti1-Δlinker
Fit EOM
χ^2 = 0.05
Sti1-ΔTPR1
Fit EOM
χ^2 = 0.77
Sti1-ΔDP2
Fit EOM
χ^2 = 0.38
Sti1-ΔTPR1DP1
Fit EOM
χ^2 = 0.03
Sti1-TPR1DP1
Fit EOM
χ^2 = 0.40
Sti1-TPR2BDP2
Fit EOM
χ^2 = 0.19
I(s) [a.u.]
s [nm^{-1}]
Residual

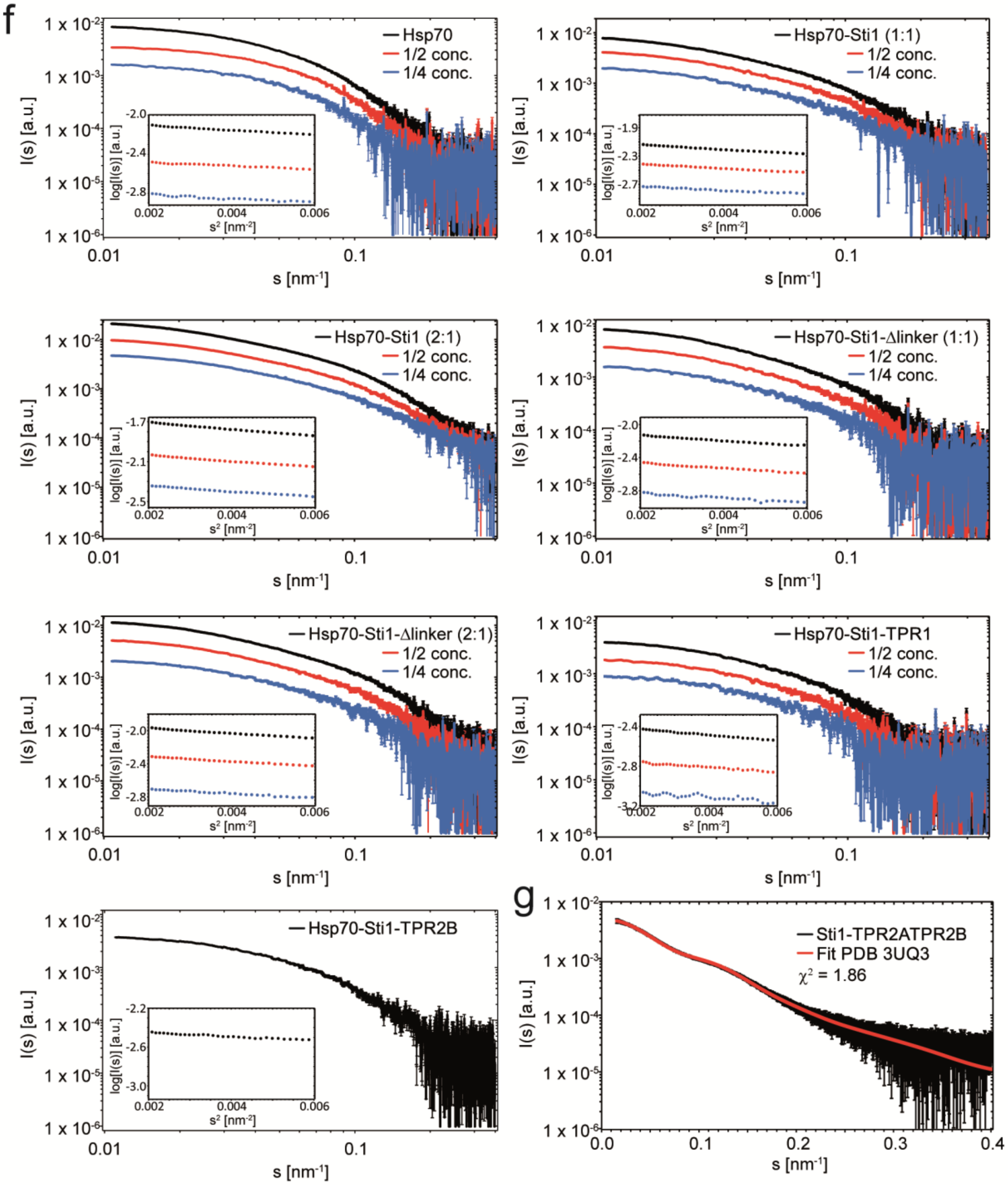
f
Hsp70
1/2 conc.
1/4 conc.
Hsp70-Sti1 (1:1)
Hsp70-Sti1 (2:1)
Hsp70-Sti1-Δlinker (1:1)
Hsp70-Sti1-Δlinker (2:1)
Hsp70-Sti1-TPR1
Hsp70-Sti1-TPR2B
I(s) [a.u.]
s [nm-1]
log[I(s)] [a.u.]
s2 [nm-2]
g
Sti1-TPR2ATPR2B
Fit PDB 3UQ3
χ2 = 1.86

Supplementary Figure 2: SAXS scattering curves and structural ensembles (a) SAXS data showing a comparison of the experimental radial density distributions of Sti1 and Sti1 fragments. **(b)** SAXS data showing a comparison of the experimental radial density distributions of Hsp70 alone and in complex with TPR1 or TPR2B. **(c)** Structural ensembles of full-length Sti1 and fragments. Color codes of the titles correspond to panel a. Multiple conformations are shown (number of structures in the ensemble in brackets), in which the TPR domains are shown in blue, the DP domains in green and linker residues in gray. Structures are aligned to TPR2A-TPR2B (Sti1, Sti1-Δlinker, ΔTPR1, ΔDP2, TPR2A-TPR2B-DP2), TPR1 (TPR1-DP1), and TPR2B (TPR2B-DP2), respectively. **(d)** Experimental X-ray scattering data of Sti1 constructs recorded at different sample concentrations in the range 1-10 mg/ml. Both the s, and I(s) axes are shown in a logarithmic representation. The angular ranges from 0.0012 - 0.4 nm^{-1} are compared. **(e)** Comparison of experimental SAXS data with SAXS data back-calculated from the EOM models. I(s) axis is shown in a logarithmic representation. The angular ranges from 0.0012 - 0.4 nm^{-1} are compared. The χ^2 of the fits and the residuals, given by $(I_{i,calc} – I_{i,obs})/err_i$, are shown as inserts. **(f)** Experimental X-ray scattering data of Hsp70-Sti1 complexes recorded at different sample concentrations in the range 1-10 mg/ml. Both the s, and I(s) axes are shown in a logarithmic representation. The angular ranges from 0.0012 - 0.4 nm^{-1} are compared. **(g)** Comparison of experimental SAXS data with SAXS data back-calculated for the TPR2ATPR2B crystal structure (PDB 3UQ3). I(s) axis is shown in a logarithmic representation. The angular ranges from 0.0012 - 0.4 nm^{-1} are compared.

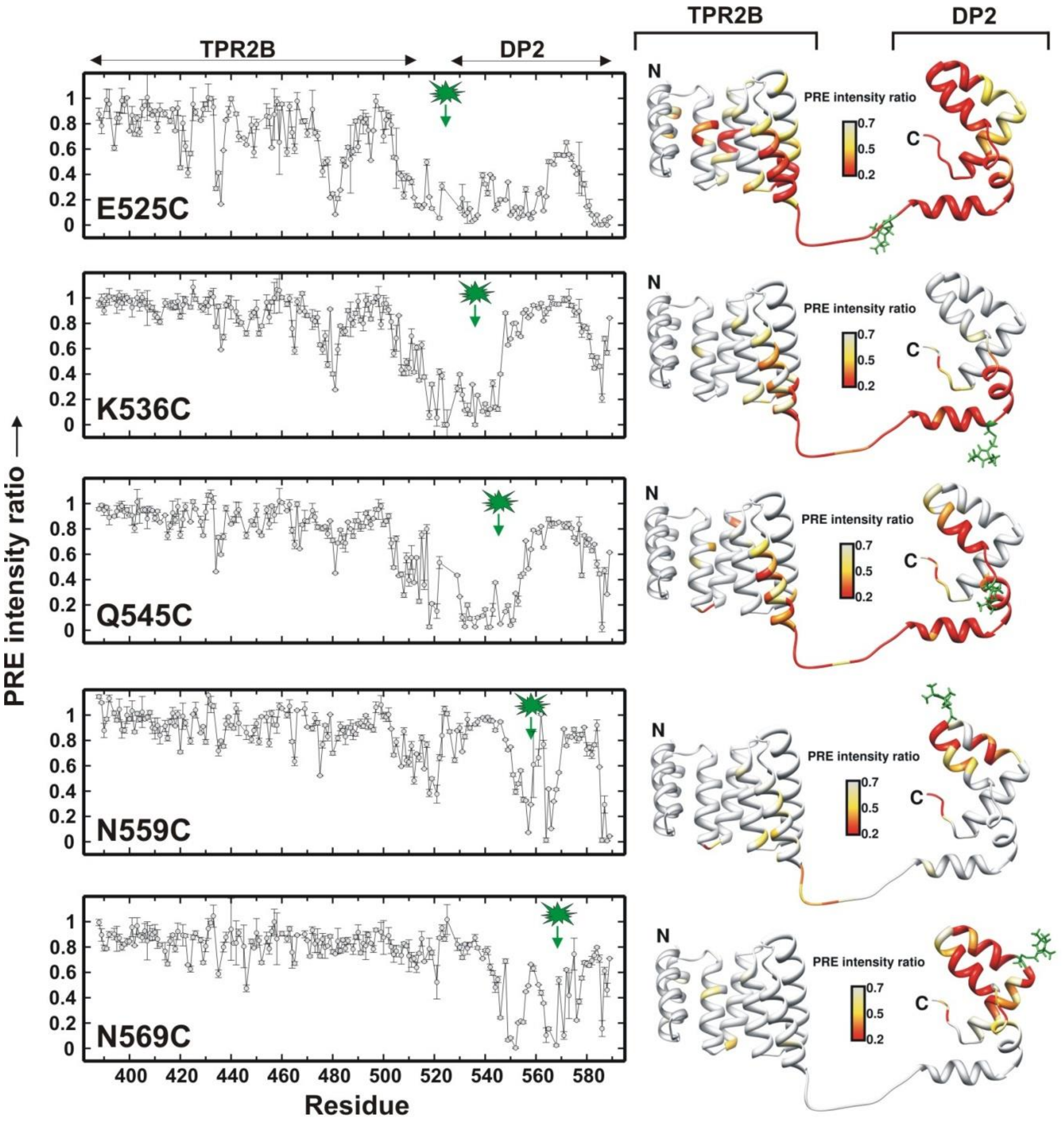

Supplementary Figure 3: Spin label NMR experiments. PRE data for the interaction between TPR2B and different PROXYL labeled DP2 variants in the two-domain construct and mapping of the positions onto a model of TPR2B-DP2. The position of the spin label is indicated in green.

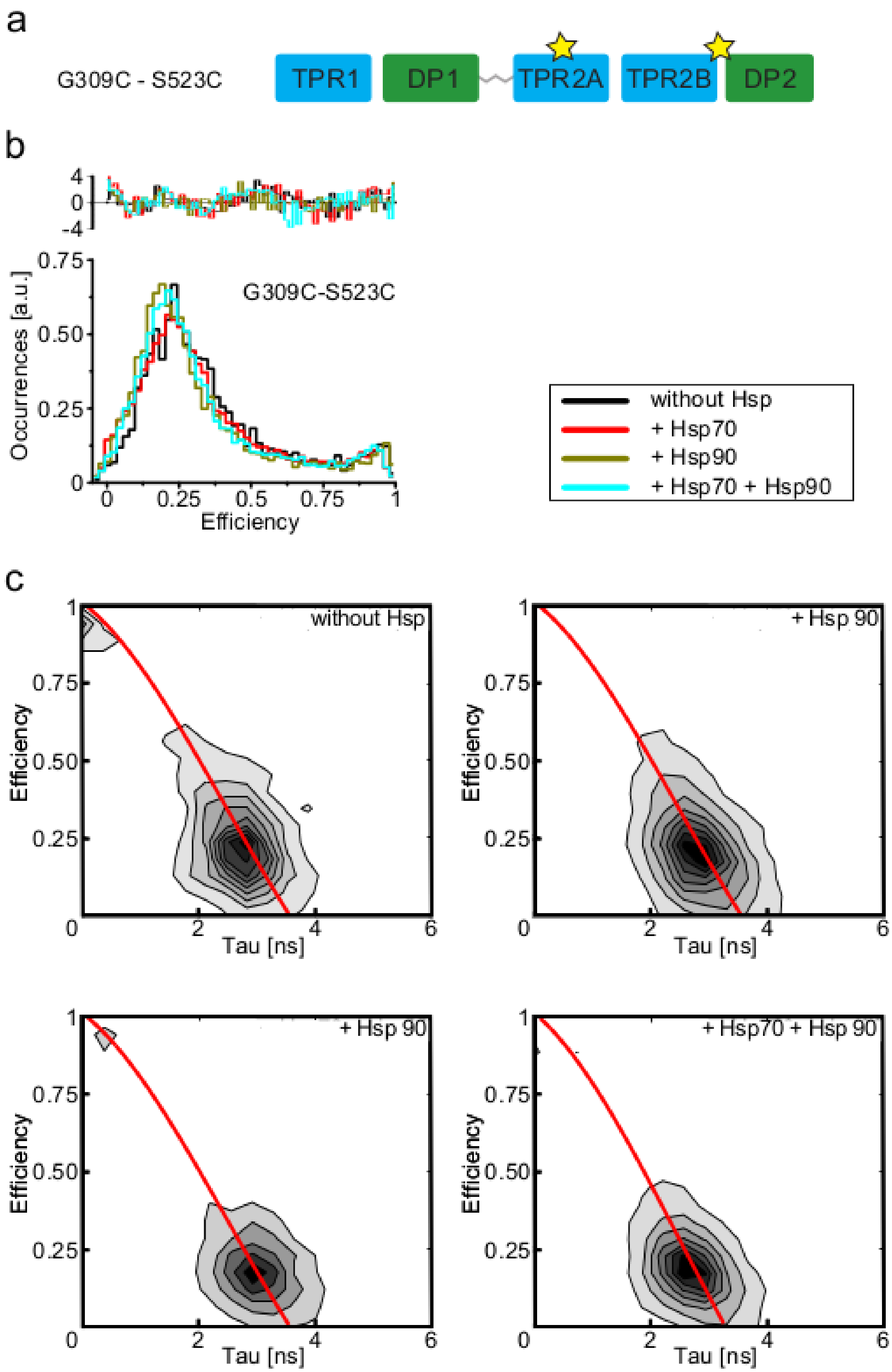
a
G309C - S523C
TPR1
DP1
TPR2A
TPR2B
DP2
b
G309C-S523C
Occurrences [a.u.]
Efficiency
without Hsp
+ Hsp70
+ Hsp90
+ Hsp70 + Hsp90
c
without Hsp
+ Hsp 90
+ Hsp 90
+ Hsp70 + Hsp 90
Efficiency
Tau [ns]

Supplementary Figure 4: Investigations of the TRP2A-TPR2B-DP2 module using spFRET. (a) Overview the double cysteine mutant discussed in this figure. The star marks the position of the cysteines, which were stochastically labeled with maleimide derivatives of ATTO532 and ATTO647. **(b)** In the lower panel the spFRET efficiency plot of the mutant in panel (a) is shown. 20 pM Sti1 was measured alone or mixed together with 10 µM Hsp90 or 25 µM Hsp70 or both. The areas under the curves were normalized to one. In the upper panel the differences between the fit of the Photon-Distribution-Analysis (PDA) and the measured data is plotted. **(c)** A burst analysis of the fluorescence lifetime of the donor in the present of the acceptor vs. the intensity calculated FRET efficiency for the G309C-S523C mutant. The data indicate a single, well-defined conformation for this construct.

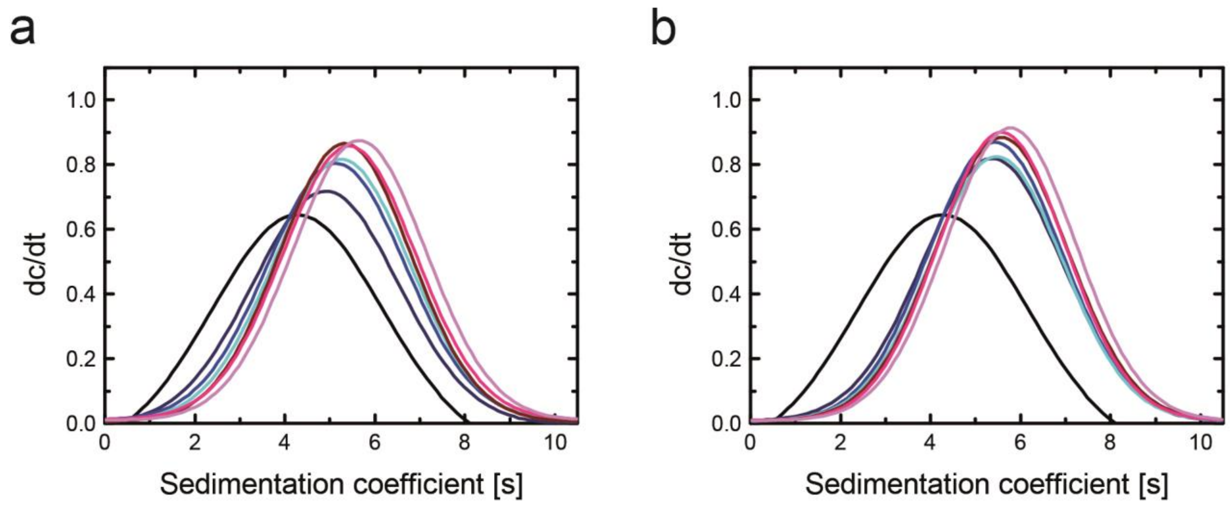

Supplementary Figure 5: Formation of binary complexes between yHsp70 and (a) Sti1 and (b) Sti1 linker deletion visualized by analytical ultracentrifugation. 0.5 µM fluorescein-labeled yHsp70 (alone: black) and in the presence of 0.5 µM (navy), 1 µM (blue), 2 µM (cyan), 3 µM (wine), 4 µM (pink) and 6 µM Sti1 (light magenta) variant in 10 mM potassium phosphate at pH 7.5. Centrifugation was performed at 20°C and 42000 rpm. Sedimentation profiles were converted into dc/dt plots according to standard procedures and fitted with Gaussian functions. For clarity, only fits are shown, standards errors were below 1%.

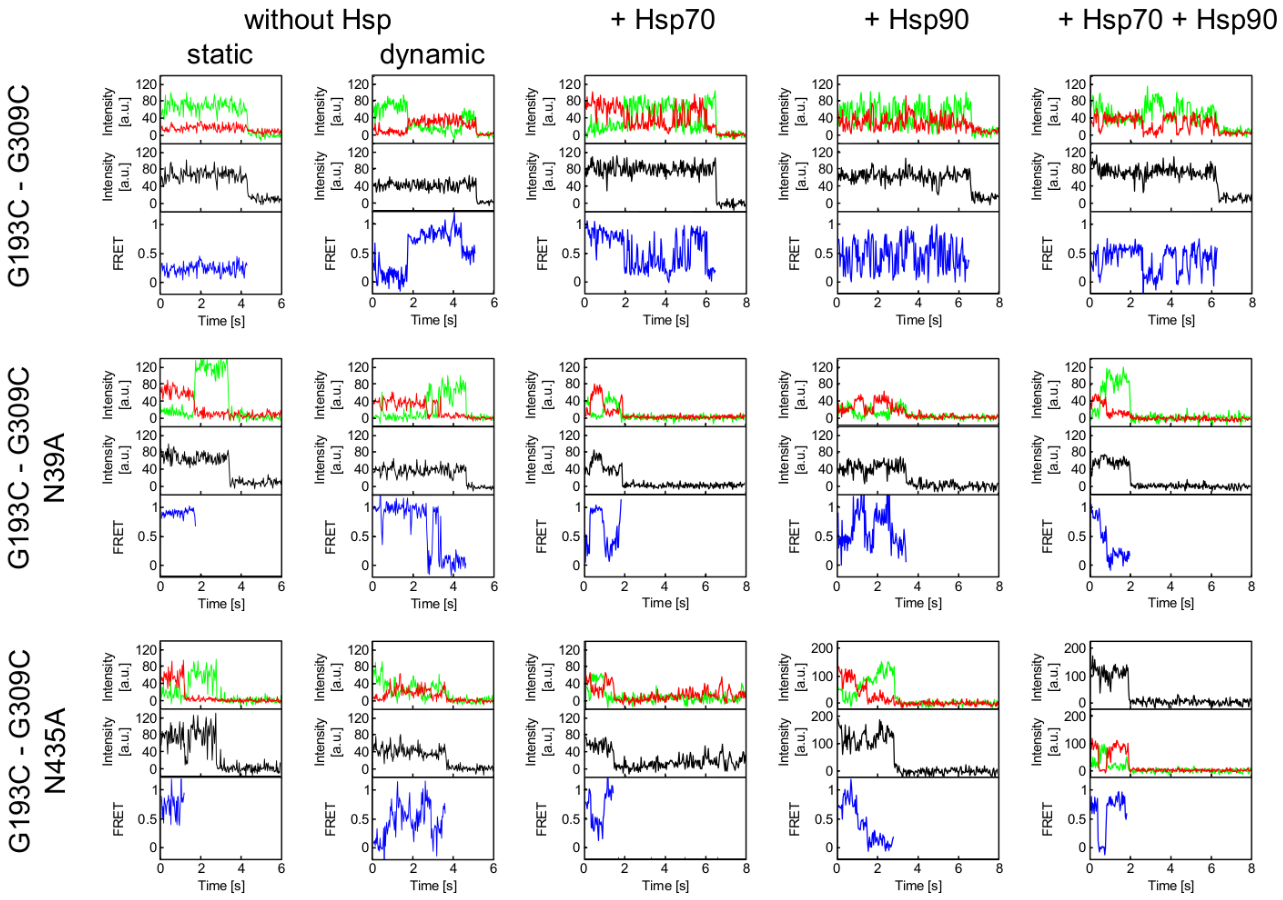

Supplementary Figure 6: Representative data from spFRET TIRF experiments. Time traces of the donor (green) and acceptor intensity (red, upper graphs), total intensity ($(I_T = \gamma I_D + I_A$ where γ is the detection correction factor, black, middle graphs) and FRET efficiency (blue, lower graphs) of the G193C-G309C, G193C-G309C N39A and G193C-G309C N435A Sti1 mutants in the absence and presence of Hsp70, Hsp90 and/or Hsp70 and Hsp90. The leftmost column represents molecules that do not show any conformational changes during the measurement. In the next column are representative spFRET traces for the different Sti1 mutant in the absence of Hsps. The middle column shows spFRET traces in the presence of 25 µM Hsp70. The fourth column show spFRET traces measured in the presence of 10 µM Hsp90 and, in the last column, spFRET traces in the presence of 25 µM Hsp70 and 10 µM Hsp90 are shown. A dynamic FRET signal is detectable in a significant fraction of the measured molecules. The fluctuations in the donor and acceptor fluorescence intensities indicate changes in the FRET efficiency due to movement of Sti1 between different conformations. For some of the traces, the donor fluorophore photobleached before the acceptor molecule and the total intensity dropped to background levels.

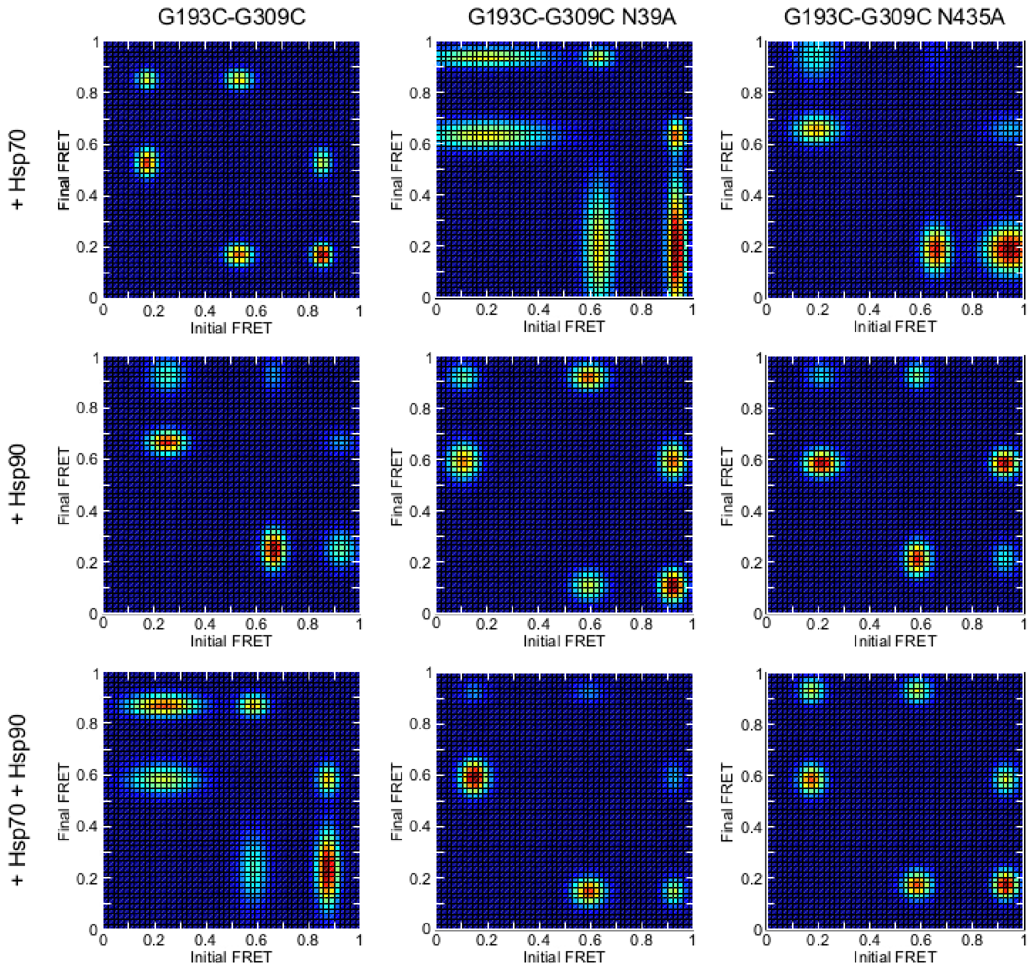

Supplementary Figure 7: Transition Density Plots from the dynamic spFRET TIRF experiments. Transition density plots of the different states of G193C-G309C, G193C-G309C N39A and G193C-G309C N435A Sti1 mutants in the presence of Hsp70, Hsp90 and/or Hsp70 and Hsp90. To generate these plots, the data were fitted with a global HMM with 3 states. The FRET efficiency and broadening of the different transitions in the *x* and *y* direction are given by the HMM analysis. The width of the peaks in the transition density plot is the width (standard deviation) of the respective FRET state given by the HMM analysis. The first row shows the plot for the mutants G193C-G309C, G193C-G309C N39A and G193C-G309C N435A in the presence 25 µM Hsp70. The next row is measured in the presence of 10 µM Hsp90 and the last row is in the presence of 25 µM Hsp70 and 10 µM Hsp90.

Supplementary Table 1. Data-collection and analyses.

Data-collection parameters	
Instrument	SAXSess mc^2 (Anton Paar)
Beam geometry	10 mm slit
Wavelength (Å)	1.5418
q range (nm^{-1})	0.012 - 0.63
Exposure time (min)	90-180
Concentration range (mg/ml)	1–10
Temperature (K)	283
Software employed	
Primary data reduction	SAXSquant (version 3.9)
Data processing	GIFT, GNOM
Rigid-body modelling	CORAL
Ensemble modeling	EOM
Three-dimensional graphics representations	PyMOL
